# Empowering Anxious Parents to Manage Child Avoidance Behaviors: Randomized Control Trial of a Single-Session Intervention for Parental Accommodation

**DOI:** 10.2196/29538

**Published:** 2021-07-06

**Authors:** Jenna Y Sung, Emma Mumper, Jessica Lee Schleider

**Affiliations:** 1 Department of Psychology Stony Brook University Stony Brook, NY United States

**Keywords:** acceptability, adolescent mental health, adolescent, anxiety, avoidance, behavior, child mental health, children, digital mental health, intervention, mental health, parent, prevention, young adult

## Abstract

**Background:**

A majority of youth who need anxiety treatment never access support. This disparity reflects a need for more accessible, scalable interventions—particularly those that may prevent anxiety in high-risk children, mitigating future need for higher-intensity care. Self-guided single-session interventions (SSIs) may offer a promising path toward this goal, given their demonstrated clinical utility, potential for disseminability, and low cost. However, existing self-guided SSIs have been designed for completion by adolescents already experiencing symptoms, and their potential for preventing anxiety in children—for instance, by mitigating known anxiety risk factors—remains unexplored.

**Objective:**

This trial evaluated the acceptability and proximal effects of project EMPOWER: a web-based, self-guided SSI designed to reduce parental accommodation, a parenting behavior known to increase the risk of anxiety in offspring.

**Methods:**

In total, 301 parents who reported elevated anxiety symptoms with children aged 4-10 years received either project EMPOWER or an informational control (containing psychoeducational materials and resources); parents self-reported their accommodation of child anxiety and overall distress tolerance at baseline and 2-week follow-up.

**Results:**

Relative to control-group parents, those who received the intervention outlined in project EMPOWER reported significant reductions in their accommodation of child anxiety (*d_s_*=0.61; *P*<.001) and significant increases in their distress tolerance (*d_s_*=0.43; *P*<.001) from baseline to 2-week follow-up. Additionally, parents rated project EMPOWER as highly acceptable (ie, easy to use, helpful, and engaging) in accordance with preregistered benchmarks.

**Conclusions:**

Project EMPOWER is an acceptable self-guided SSI for parents of children at-risk for anxiety, which yields proximal reductions in clinically relevant targets.

**Trial Registration:**

ClinicalTrials.gov NCT04453865; https://tinyurl.com/4h84j8t9

## Introduction

### Background

Anxiety disorders are among the most common, debilitating forms of childhood psychopathology, affecting 8.3%-27.0% of youth aged less than 18 years [1,2]. Child anxiety increases the risk for psychiatric comorbidities across the lifespan [3], creates significant burdens for caregivers [4], and carries stark societal costs [5,6]. Although numerous interventions have been developed to treat anxiety disorders in the youth, up to 82.2% of the youth in the United States, who have anxiety do not receive adequate care [7]. Several factors may explain this discrepancy, including the length and cost of existing treatments and limited accessibility for families in need. Together, these factors create a pressing need for accessible, brief preventive programs to decrease the odds of the onset of anxiety disorder in at-risk youth.

Single-session interventions (SSIs) may offer a potential solution to bridge this gap in care. SSIs include core components of comprehensive evidence-based interventions delivered succinctly to improve the odds of access and completion [8]. In a meta-analysis of 50 randomized trials, SSIs reduced youth psychopathology across multiple disorders, with SSIs that target child anxiety producing especially large effects (mean *g*=0.58) [8]. Thus, well-targeted SSIs may offer cost-effective additions or alternatives to traditional care for anxiety in the youth. However, most existing SSIs for child anxiety target populations already experiencing clinical distress, highlighting the requirement of options that may prevent anxiety in vulnerable children. Given that family factors play a crucial role in the etiology of child anxiety [1], SSIs targeting parents and their interactions with offspring may be a promising approach to preventing anxiety in the youth [9]. Thus, this trial examined the acceptability and short-term effects of a novel, web-based, self-guided SSI targeting parental accommodation: a well-established, potentially modifiable risk factor for child anxiety [10-13].

### Parental Accommodation as a Modifiable Intervention Target

Parental accommodation refers to changes in caregiver behaviors that facilitate or maintain their child’s anxiety-driven avoidance behaviors [14,15]. Examples of such behaviors include modifying family routines (ie, staying home from work to mitigate a child’s separation fears) or directly participating in a child’s avoidance strategies (ie, keeping a child home from school). Parental accommodation reduces children’s immediate distress but maintains their long-term avoidance of feared stimuli or situations, and high levels of parental accommodation are associated with more severe anxiety symptoms in offspring [15-17]. Parental accommodation may be further maintained by caregiver-level factors, including elevated parental anxiety symptoms and low distress tolerance. For instance, accommodation behaviors are more frequent among parents who report higher distress about their child’s anxiety symptoms [18] and perceptions that experiencing anxiety is harmful to the youth [19].

Parental accommodation can also be systematically reduced through psychosocial intervention. For instance, in trials of the 12-week, parent-directed, therapist-delivered Supportive Parenting for Anxious Childhood Emotions program, which targets parents’ accommodation, has helped mitigate anxiety in children with subclinically and clinically elevated anxiety symptoms [15,20]; recent studies even suggest that parent-directed, accommodation-focused treatments may be noninferior to exposure therapy for treating child anxiety [21]. Translating core components of existing multisession interventions for parental accommodation into briefer, self-guided SSIs (ie, those that do not involve a trained therapist) may improve families’ access to empirically driven supports. Thus, we developed and tested a web-based, self-guided SSI for parents—project EMPOWER—to provide psychoeducation and teach skills to reduce parents’ accommodation of avoidance behaviors in their school-aged children. Within an enhanced waitlist-control design, parents were randomized to either Online Resources and Referrals (ORR) and project EMPOWER (ORR+EMPOWER group) or ORR and delayed Project EMPOWER access (2 weeks after study conclusion) (ORR+waitlist group). We predicted that parents would report larger declines in self-reported accommodation behaviors (primary outcome) and larger increases in distress tolerance (secondary outcome) in the ORR+EMPOWER group, relative to the ORR+waitlist group, from baseline to 2-week follow-up. We also predicted that parents completing project EMPOWER would subjectively perceive larger pre-SSI to immediate post-SSI increases in their ability to help their child manage distressing situations, relative to control-group parents. Finally, we predicted that parents completing project EMPOWER would rate the intervention as acceptable (enjoyable, worth recommending to other parents, and personally helpful).

Notably, because this trial constituted the first formal test of project EMPOWER, the study’s primary goal was to assess the program’s potential to engage its intended mechanistic target: parental accommodation of avoidance and anxiety in their young children. If project EMPOWER can systematically improve this target in parents of children with or without clinically elevated anxiety, this study may lay the foundation for future trials on project EMPOWER’s capacity to prevent child anxiety symptoms in the longer term.

## Methods

### Ethical Considerations

Study procedures were reviewed and approved by the institutional review board of the university, and informed consent was obtained from each participant via the internet prior to participation. The trial and all methods were prospectively preregistered on in ClincialTrials.gov prior to participant enrollment (NCT04453865).

### Recruitment and the Resulting Sample

In total, 301 parents of children aged 4-10 years were recruited through Facebook advertisements, following established ethics guidelines for passive, social media–based study recruitment [22]. Participants were eligible for the study if they (1) reported subclinical or greater anxiety symptoms (a score of >40 on the Penn State Worry Questionnaire [PSWQ]) because children whose parents have high levels of anxiety are at an elevated risk for developing anxiety themselves, and parents with high levels of anxiety report engaging in more accommodation than do those with lower levels of anxiety [17]; (2) had at least 1 child aged 4-10 years; and (3) displayed comfort with English (intervention materials were available in English only). This specific child age range was selected because it encompasses the age of onset for common child anxiety disorders [23]; it also matches the age-range for which parent-focused interventions are often designed [24]. Study recruitment began in July 2020 and ended in August 2020 once the target number of participants was achieved.

### Procedures

After clicking on a social media advertisement, parents were directed to an informational study webpage that invited them to complete a web-based eligibility screener. Eligible parents then reviewed a web-based consent form that invited them to participate. Parents could initiate the study at any time and location, using any internet-equipped device (smartphone, laptop, or tablet device). After starting the study, participants first completed preintervention self-report questionnaires, which are detailed below. Within the same survey, participants were randomized via Qualtrics (1:1 allocation ratio) to receive either ORR and immediate access to project EMPOWER (intervention condition) or ORR and delayed access to project EMPOWER after the 2-week follow-up (control condition). Those in the intervention condition also completed the Program Feedback Scale, along with other postintervention surveys, immediately following the completion of project EMPOWER. Two weeks later, all parents—regardless of condition—were invited to complete follow-up questionnaires. Parents in the control condition were then invited to complete project EMPOWER, if they were interested (completion of project EMPOWER subsequent to follow-up questionnaires was optional and was not part of the study). Thus, all participants were able to complete project EMPOWER, either immediately or after a 2-week delay.

### Intervention

Project EMPOWER (freely available for anonymous completion on the project’s website [25]) is a web-based, self-guided SSI for parents, which takes 20-30 minutes to complete. The program includes 5 main elements, which are based on current recommended practices in SSI design [26] and existing, therapist-delivered interventions targeting parental accommodation [15,21]:

1. Psychoeducation on child anxiety and avoidance, along with how parental accommodation can inadvertently foster child anxiety;
2. Information on how parents can better identify children’s patterns of avoidance and encourage “brave behavior,” instead;
3. An exercise that guides parents in creating a personalized, step-by-step “action plan” for promoting brave, approach-oriented behaviors (rather than anxiety-driven avoidance) in their own child;
4. A segment intended to normalize parent distress responses in response to anxiety in offspring, including a rationale for why encouraging “brave behaviors”—despite being emotionally challenging for caregivers—ultimately bolsters children’s well-being and resilience; and
5. A vignette exercise in which parents read about another family’s difficulty managing child anxiety; parents identify various elements of the “anxiety cycle” (in accordance with psychoeducation provided previously) and generate possible solutions for the parents described in the vignette, which are based on their newfound knowledge of promoting “brave behavior” in the youth.

### Control Condition

ORR included an information sheet containing a list of web-based psychoeducational resources (videos, books, web-based toolkits, etc) on anxiety, hotlines, and resources on finding mental health treatment around the United States. ORR did not include any psychoeducational components explicitly designed to reduce parental accommodation of child anxiety. The full content of ORR is provided in Multimedia Appendix 1.

### Measures

Other measures not detailed here were included in the study for exploratory purposes. The full battery of measures included in the study can be found on the registration page on ClinicalTrials.gov.

### Demographics

Parents self-reported the gender, biological sex, ethnicity, country of origin, education level, and age for themselves and their children.

### Parental Accommodation of Child Anxiety (Primary Outcome)

Using the Family Accommodation Scale—Anxiety (FASA) [10], parents rated agreement with 9 items, which reflected the extent to which they accommodate their child’s anxiety symptoms or avoidance behaviors. Higher mean scores indicate more frequent parental accommodation. As a primary outcome measure, the FASA was administered at baseline and 2-week follow up to all participants. The FASA has demonstrated excellent psychometric properties across numerous studies [10]. Here we used *α* values of .87 and .85 at baseline and 2-week follow-up, respectively.

### Parent Distress Tolerance (Secondary Outcome)

Using the 16-item Distress Tolerance Scale (DTS) [27]—a valid, reliable measure of overall distress tolerance in adults—parents rated their perceived ability to experience and withstand distressing emotional states on a 5-point scale. Higher mean DTS scores reflect lower levels of distress tolerance. As a secondary outcome measure, DTS was administered at baseline and 2-week follow to all study participants. Here we used *α* values of .86 and .88 at baseline and 2-week follow-up, respectively.

### Child Anxiety Symptoms

Parents completed Revised Children’s Anxiety and Depression Scale-Parent Report (RCADS-25-P) [28]: a well-validated, 25-item measure that assesses child internalizing symptoms. Parents endorsed the presence (or absence) of 25 different anxiety and depressive symptoms in children, each on a 4-point scale. Higher scores reflect more severe child internalizing symptomatology. The RCADS-25-P was completed at baseline only to characterize the level of anxiety experienced by children of participating parents. Here we used an *α* value of .85. Notably, we did not assess child anxiety at 2-week follow-up in this study, because the trial’s objective was to establish whether project EMPOWER could successfully engage its intended target (parental accommodation behaviors).

### Parent Anxiety Symptoms

Parents completed the PSWQ [29]—a well-validated 16-item self-report questionnaire that asks respondents to rate their perceived experience of worry- and anxiety-related problems using a 5-point scale. Higher sum scores indicate more severe worry. Parents completed the PSWQ at baseline to screen for subclinical or higher parental anxiety levels (an inclusion criterion) and to characterize the participating parent sample by anxiety severity. The PSWQ has demonstrated high internal consistency and good test-retest reliability [29]. Here we used an *α* value of .88.

### Perceived Change in Preparedness to Help Children Manage Distress

A single-item measure that gauges participants’ perceived changes in their ability to help their children manage distressing situations was adapted for this study [26]. All participants were asked to rate their agreement with a single-item statement on a 5-point scale, either immediately after completing project EMPOWER (intervention condition) or immediately after being presented with psychoeducational materials (informational waitlist condition): “Compared to before you started this survey, how prepared do you feel to help your child manage distressing situations?” This item was administered immediately post SSI only for the intervention group as a secondary exploratory outcome.

### Intervention Acceptability

Parents in the intervention condition completed the Program Feedback Scale (PFS) [30]—a reliable and valid measure routinely used to assess acceptability and user perceptions of web-based, self-guided SSIs. The PFS asks participants to rate 7 statements on a 5-point scale (scores ranging 1-5) and share what they liked and what they would change about the SSI, in an open-response format. A mean score of ≥3 indicates acceptability and positive program evaluation. The PFS was administered post SSI to parents assigned to the intervention condition to assess program acceptability.

### Power Analysis

Using G*Power (version 3.1, Heinrich-Heine-Universität Düsseldorf), sample sizes needed to detect group differences in the primary outcome (changes in accommodation from baseline to follow-up) between the intervention and control groups of small (.2), medium (.5), and large effects (.8) based on an *F*-test, linear multiple regression with *α*=.05, and power=0.80, were 395, 55, and 25, respectively. Thus, our sample (n=301) offered sufficient power to detect a small-to-medium between-groups effects (consistent with effect sizes observed in previous randomized trials on web-based SSIs) [31].

### Missing Data

We imputed missing data using the expectation maximization and bootstrapping algorithms implemented with Amelia II in R, as no evidence emerged for unequal drop-out by condition. These imputed data sets allowed for more conservative intent-to-treat analyses than listwise deletion or last-observation carried forward and allowed us to retain high power even considering missing data. We imputed 60 data sets in accordance with the proportion of missing data for our primary outcome measure (using FASA) at 2-week follow-up.

### Analysis Plan

The entire preregistration can be found on ClinicalTrials.gov (NCT04453865). Deidentified data and code for all preregistered analyses are available on the Open Science Framework [32].

### Effects of Project EMPOWER on Primary and Secondary Outcomes

To assess the effects of the intervention on parent’s accommodation levels and distress tolerance from baseline to 2-week follow up, we used a multiple linear regression approach with the intervention condition (1=ORR+EMPOWER; 0=ORR+waitlist), baseline accommodation levels, and parental distress as predictor variables to examine whether participants in each condition saw a differential reduction in outcome variables. Using the MOTE R Package, we also reported the Cohen *d* effect sizes and 95% CIs for within-group (*d_*av; reflecting intervention effects for changes before the intervention up to follow-up) and between-group (*d_*s; reflecting changes in outcome before the intervention up to follow-up in the 2 groups) differences in both accommodation and distress tolerance levels [33].

### Perceived Change in Preparedness to Manage Child Anxiety Before and After the Intervention

A 2-sample *t* test was performed to determine whether the overall, subjectively detectable pre-to-post changes in “preparedness to help their child manage anxiety” significantly differed between parents who completed project EMPOWER immediately, compared to control-group parents.

### Project EMPOWER Acceptability

We examined overall and item-level mean PFS scores among parents who completed project EMPOWER. Mean and item-level scores of >3 or higher on any item (on a 5-point scale) reflected the endorsement of the program’s acceptability (eg, positive feedback), either for that specific item or overall.

### Project EMPOWER Completion Rates

**Figure 1:**
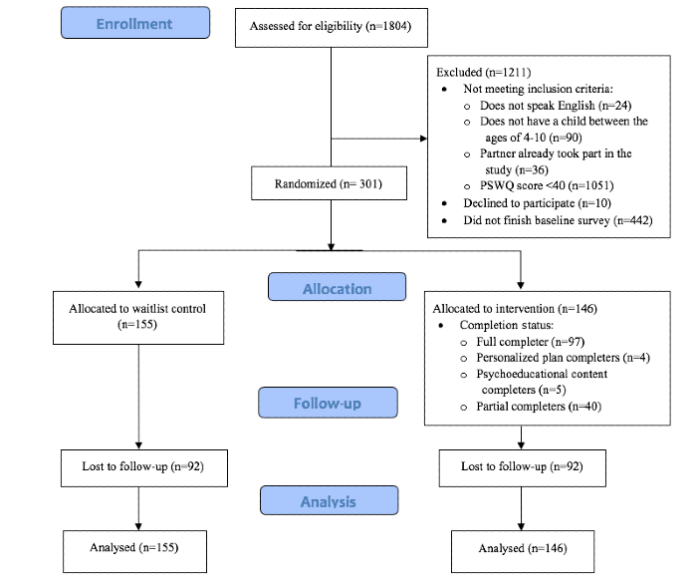
CONSORT (Consolidated Standards of Reporting Trials) diagram. PSWQ: Penn State Worry Questionnaire.

Operational definitions of differential “program completer” status among parents assigned to the project EMPOWER condition were preregistered prior to data analysis. Full completers were those who reached the final page of the intervention, thus receiving the full “dose” of intended materials (approximate completion time: 25-30 minutes); personalized plan completers completed all psychoeducational content in project EMPOWER and finished their personalized plan for promoting brave behavior in their child (approximate completion time: 20-25 minutes); psychoeducational content completers completed all psychoeducational content, but not a personalized plan (approximate completion time: 10-15 minutes); and partial completers began the intervention but did not reach any of the above-mentioned program benchmarks. We report completion rates at each level, among parents assigned to the project EMPOWER condition, in the CONSORT diagram (Figure 1).

## Results

### Sample Characteristics

Characteristics of the 301 participating parents and their children are shown in Table 1. Parents were predominantly female (98.00%), 67.77% were White, 9.63% were Asian, 9.63% were of other racial backgrounds, 6.31% were Hispanic/Latino/a, 2.33% were American Indian/Alaska native, 1.99% were of more than 1 ethnicity, 1.66% were Black/African American, and 0.66% were native Hawaiian/Pacific Islanders; 52.82% had a received a graduate or professional degree. Consistent with our goal to recruit parents with elevated levels of anxiety, participants reported a mean score of 60.74 (SD 9.84) on the PSWQ, immediately below the clinical cut-off score of 62, in line with Behar et al [34]. Parents indicated a broad range of difficulties facing their child (raw RCADS score 18.83, SD 9.95), which included the following: clinically significant anxiety symptoms (76.74%), mood problems (17.94%), behavioral problems (33.89%), attention problems (33.89%), developmental delay (6.31%), learning disabilities (7.31%), peer relationships (35.55%), family relationship problems (21.93%), and trauma (15.28%).

**Table 1:** Sample characteristics.

Variable				Project EMPOWER (n=146)		Waitlist control (n=155)
Parents’ score on the Penn State Worry Questionnaire, mean (SD)				60.30 (9.81)		61.18 (9.89)
Youths’ score on the Revised Children’s Anxiety and Depression Scale-Parent Report, mean (SD)				18.08 (9.64)		19.53 (10.22)
Age of the youths (years), mean (SD)				6.77 (1.93)		6.73 (2.03)
Female youths, n (%)				75 (51.02)		67 (43.51)
**Race and ethnicity of the youths, n (%)**						
	American Indian/Alaska Native		5 (3.42)		5 (3.23)	
	Asian		11 (7.53)		11 (7.10)	
	Black/African American		4 (2.74)		1 (0.65)	
	Hispanic/Latino/a		6 (4.11)		8 (5.16)	
	White/Non-Hispanic		87 (59.59)		101 (65.16)	
	>1 Race		14 (9.59)		19 (12.26)	
	Other		19 (13.01)		10 (6.45)	
**Annual family income (US $), n (%)**						
	0-19,000		9 (6.16)		8 (5.16)	
	20,000-39,000		16 (10.96)		20 (12.90)	
	40,000-59,000		18 (12.33)		12 (7.74)	
	60,000-79,000		16 (10.96)		18 (11.61)	
	80,000-99,000		16 (10.96)		15 (9.68)	
	100,000-119,000		11 (7.53)		14 (9.03)	
	120,000-140,000		15 (10.27)		13 (8.39)	
	>140,000		27 (18.49)		31 (20.00)	
**Marital status, n (%)**						
		Married		104 (71.23)		118 (76.13)
		Living with partner		14 (9.59)		12 (7.74)
		Never married		13 (8.90)		10 (6.45)
		Divorced		9 (6.16)		8 (5.16)
		Separated		4 (2.74)		6 (3.87)
		Widowed		2 (1.37)		1 (0.65)
		Single parent		32 (21.92)		32 (20.65)
Number of children, mean (SD)				2.02 (1.00)		2.19 (1.20)
Female parents, n (%)				143 (97.95)		152 (98.06)

### Did Project EMPOWER Reduce Parental Accommodation of Anxiety and Improve Parent Distress Tolerance?

Parents assigned to the project EMPOWER condition reported significantly greater reductions in the accommodation of their children’s anxiety (between-group *d_s_*=0.61; *P*<.001), as well as significantly greater improvements in distress tolerance (*d_av_*=0.17; between-group *d_s_*=0.43; *P*<.001) from baseline to 2-week follow-up, relative to control-group parents. Table 2 provides additional details regarding the multiple linear regression approach.

Regarding within-group effects, parents who participated in project EMPOWER reported significant 2-week reductions in accommodation of child anxiety (project EMPOWER within-group *d_av_*=0.67), whereas those who were assigned to the control condition did not (control within-group *d_av_*=0.17). Between- and within-group effect sizes (*d_av_* and *d_s_*) and 95% CIs are reported in Table 3.

**Table 2:** Results of multiple linear regression analysis in predicting intervention effects on parental accommodation (using FASA^a^) and distress tolerance (using DTS^b^) at 2-week follow-up.

Parameter	Parent-reported accommodation		Parent-reported distress tolerance	
	Coefficient (SE)	*P* value	Coefficient (SE)	*P* value
FASA score at baseline	0.53 (0.07)	<.001	N/A^c^	N/A
DTS score at baseline	N/A	N/A	0.77 (0.07)	<.001
Intercept	0.79 (0.15)	<.001	0.75 (0.20)	<.001
Condition	–0.48 (0.11)	<.001	–0.24 (0.09)	.008

^a^FASA: Family Accommodation Scale—Anxiety.

^b^DTS: Distress Tolerance Scale.

^c^N/A: not applicable.

**Table 3:** Means, standard deviations, and effect sizes of outcome variables by condition.

Outcome variable	Project EMPOWER		Cohen d_av^a^ (95% CI)	Control group		Cohen d_av (95% CI)	Cohen d_s^b^ (95% CI)
	Mean (SD) at baseline	Mean (SD) at 2-week follow-up		Mean (SD) at baseline	Mean (SD) at 2-week follow-up		
Score on the Family Accommodation Scale—Anxiety	1.83 (0.91)	1.29 (0.71)	0.67 (0.49-0.85)	1.88 (0.90)	1.79 (0.81)	0.11 (–0.05-0.26)	0.61 (0.38-0.84)
Score on the Distress Tolerance Scale	2.77 (0.76)	2.64 (0.73)	0.17 (0.01-0.34)	2.73 (0.72)	2.85 (0.76)	–0.16 (–0.32-0.00)	0.43 (0.20-0.66)

^a^Cohen *d*_av reflects within-group changes in each outcome variable.

^b^Cohen *d*_s reflects between-group changes in each outcome variable.

### Did Parents who Completed Project EMPOWER Perceive Improvements in Their Preparedness to Manage Child Distress?

Immediately following the completion of either the control condition or project EMPOWER, participants were asked the following question: “Compared to before you started this survey, how prepared do you feel to help your child manage distressing situations?” On a scale of 1 (“much less prepared to help my child”) to 5 (“a lot more prepared to help my child”), parents who completed project EMPOWER reported feeling significantly more prepared to help their child than those in the control group (*t_155.27_*=8.66; *P*<.001). Among parents who received immediate access to project EMPOWER and completed the intervention, 54.28% reported feeling “a little more prepared to help my child,” 30.00% reported feeling “a lot more prepared,” and 15.71% reported feeling “the same amount prepared.” No participant reported feeling less prepared to help their child.

### Was Project EMPOWER Acceptable?

Among parents who were assigned to the intervention condition, the majority (n=97, 66.44%) fully completed project EMPOWER, 4 (2.74%) qualified as personalized plan completers, 5 (3.42%) were psychoeducation content completers, 32 (21.92%) were partial completers, and the remaining parents (5.48%) did not begin project EMPOWER after randomization. Parents who completed project EMPOWER rated the intervention as acceptable in accordance with a mean PFS score of 4.25 of 5.00 (higher than the preregistered cut-off score of 3.00). More specifically, parents rated the intervention as easy to understand (4.41 of 5.00), easy to use (4.31 of 5.00), likely to help other parents (4.31 of 5.00), enjoyable (3.92 of 5.00), worth recommending to other parents (4.20 of 5.00), and endorsed agreement with the program’s message (4.56 of 5.00).

## Discussion

### Principal Findings

Our results support the short-term efficacy and acceptability of project EMPOWER: a self-guided, web-based SSI designed to reduce parental accommodation of child anxiety. Compared to a psychoeducational control, project EMPOWER yielded significant reductions in clinically relevant outcomes—parental accommodation and distress tolerance—across a 2-week follow-up period. Additionally, participating parents viewed project EMPOWER as highly acceptable and subjectively helpful for managing their child’s distress relative to the psychoeducational control. Moreover, parents who began project EMPOWER completed the program at a relatively high rate (66.44%), both when compared to a prior naturalistic program evaluation of web-based, self-guided SSIs (eg, 34.32% completion rates for 3 other web-based SSIs) [26] and compared to completion rates reported for similar self-guided, digital mental health support tools (0.5%-28.6%) [35]. This retention level within a self-guided program suggests project EMPOWER’s strong acceptability among its users. Together, our results suggest the promise of project EMPOWER to mitigate known risk factors for anxiety in children, and specifically those with parents who have high levels of anxiety.

Notably, the between-group effects of project EMPOWER on parental accommodation (*d_FASA_*=0.61) compared favorably to the effects observed in separate trials of treatments targeting parental accommodation—including those observed in a trial of a 12-week, parent-directed, therapist-guided intervention (the Supportive Parenting for Anxious Childhood Emotions program [15], postintervention *d_FASA_*= 0.22 vs child-directed exposure therapy). The effect sizes in project EMPOWER also compare favorably to previously reported effects of SSIs that directly target anxiety in the youth (post-SSI *d_child anxiety_*=0.56) [8]. These previously observed effects serve as approximate benchmarks for the impact of project EMPOWER, rather than direct comparisons, owing to variation in the methods (eg, more vs less active comparison groups), follow-up duration, and intervention intensity. Nonetheless, our results are the first to suggest that a 30-minute, fully self-guided, parent-directed intervention may help reduce parental accommodation, potentially helping to mitigate anxiety in their children. Frequently cited benefits of self-guided, web-based SSIs—including their potential for rapid scalability, their free availability to users, and the ability to complete them at any time and location [26,31]—highlight the high potential public health impact of project EMPOWER, catering to individuals and populations who may otherwise be unable to access support.

### Limitations

Several limitations of this trial warrant discussion and suggest directions for future studies. First, although the completely web-based study design allowed for a large sample size and rapid, low-cost recruitment through social media, the lack of monetary compensation likely contributed to substantial attrition at follow-up (61.13%), despite scheduled email reminders. However, it is worth noting that offering greater monetary compensation may have introduced additional bias to the sample selection. This limitation was addressed via a rigorous missing data approach, which has shown utility with high rates of missing data, including those observed in this trial [36]. Second, similar to the limitations noted in much of the literature on parenting interventions, the homogeneity in sex (98% mothers), race and ethnicity, and education status in our sample limited the generalizability of our results across diverse groups of parents. This may be due to the selection bias introduced by recruitment through social media as Facebook likely distributed the advertisements to users who are interested in the study topic. As the study team did not have control over the algorithms that are used to distribute the advertisement, it limited our ability to reach a more diverse population. Moving forward, it will be critical to test the acceptability and effects of project EMPOWER and other self-guided SSIs among members of marginalized and minoritized communities of individuals who are systematically least likely to access traditional, face-to-face mental health treatments owing to financial, logistic, and stigma-related barriers.

Given that non–English-speaking parents were unable to take part in the study (project EMPOWER is currently available only in English), efforts to translate project EMPOWER into various languages may greatly facilitate tests of its acceptability and utility among more diverse caregivers. Third, because this trial was the first to assess the acceptability and proximal effects of project EMPOWER, we included a relatively brief 2-week follow-up period. Thus, results address only the short-term effects of the intervention on known risk factors for child anxiety. Given that some trials of self-guided SSIs have demonstrated clinical benefits for youth up to 9 months after the intervention [31,37], the longer-term effects of project EMPOWER remain important to explore. Such studies may investigate whether the intervention can prevent the emergence of child anxiety symptoms and evaluate improvements in parental accommodation and distress tolerance as possible change mechanisms.

### Future Directions

Future studies should examine whether and how project EMPOWER may be useful as both a standalone intervention (as assessed here) and as a possible adjunctive support in the context of longer-term, child-directed anxiety treatment, for families in need of more intensive clinical support. For example, clinicians may assign project EMPOWER as “homework” to augment traditional psychoeducation about the nature and maintenance of anxiety; alternatively, therapists might deploy project EMPOWER as a relapse prevention tool, which would be introduced upon termination of child-focused treatment or as an interim support for families on waiting lists for treatment. Future studies should gauge the potential of project EMPOWER as a therapy-augmenting tool and evaluate its impact on treatment duration and symptom changes in the youth. Given the potential of project EMPOWER for rapid scalability (as a free, self-guided, web-based intervention), future studies should also evaluate its possible use across multiple settings and among diverse populations. This initial trial recruited the parents of young children; however, project EMPOWER teaches skills of potential relevance to any adult who interacts with children, including teachers, mentors, and health care workers. Therefore, project EMPOWER may be integrated into numerous existing environments of the youth through direct distribution to adults who care for them, who may utilize the program however and wherever they choose.

### Conclusions

In conclusion, project EMPOWER shows promise as a scalable, brief, self-guided approach to reducing accommodation behaviors and strengthening distress tolerance among parents of school-aged children, who have high levels of anxiety—at least over the short term.

## Appendix

Multimedia Appendix 1 Control (ORR) Condition - Online Resources and Referrals, Project EMPOWER.

Multimedia Appendix 2 CONSORT-eHEALTH checklist (V.1.6.1).

## Abbreviations

DTS: Distress Tolerance Scale
FASA: Family Accommodation Scale—Anxiety
ORR: Online Resources and Referrals
PFS: Program Feedback Scale
PSWQ: Penn State Worry Questionnaire
RCADS-25-P: Revised Children’s Anxiety and Depression Scale-Parent Report
SSI: single-session interventions
