# Supplementary material for: Empowering Anxious Parents to Manage Child Avoidance Behaviors: Randomized Control Trial of a Single-Session Intervention for Parental Accommodation
Source: JMIR Ment Health. 2021 Jul 6;8(7):e29538. doi: 10.2196/29538 (PMC8292931; doi:10.2196/29538)
Supplement: Multimedia Appendix 1 [file mental_v8i7e29538_app1.pdf]

WELL-BEING & MENTAL HEALTH RESOURCE LIST: YOUTH FRIENDLY MENTAL HEALTH ONLINE RESOURCES FOR CAREGIVERS

AACAP FAMILY RESOURCE CENTERS

Geared towards parents, this website provides learning tools on a variety of mental illnesses, videos, and resources for kids.

KELTY MENTAL HEALTH RESOURCE CENTER

Reference sheets are provided that list top websites, books, videos, toolkits and support for mental health disorders.

ADAA TIPS FOR PARENTS AND CAREGIVERS

This website provides tips for parents and caregivers to support a child with anxiety

NASP INFORMATON FOR PARENTS

This website contains information about anxiety disorders, as well as resources to help parents identify anxiety symptoms in young children.

CHILD MIND INSTITUTE RESOURCES FOR FAMILIES

This website provides information on specific mental health disorders, as well as resources to help children use mindfulness and cope with life stressors.

COPING SKILLS FOR KIDS

Coping Skills for Kids provides products and resources for children to cope with stress, anxiety, and anger

POSITIVE PSYCHOLOGY MINDFULNESS ACTIVITIES

This website provides activities to help children to use mindfulness to handle stress and improve mental wellbeing.

NATIONAL PARENT HOTLINE

Call 1-855-427-2736 to talk with a trained parent advocate to help caregivers problem-solve and offer emotional support

WELL-BEING & MENTAL HEALTH RESOURCE LIST: TREATMENT REFERRALS FOR YOUTH AND CAREGIVERS

PSYCH CENTRAL CHOOSING A THERAPIST

This website has tips to help caregivers search for services and choose the right therapist for their child

SCCAP EFFECTIVE CHILD THERAPY

This website provides a guide for caregivers about evidenced-based mental health treatments for youth

PSYCHOLOGY TODAY RESOURCES TO FIND A CHILD THERAPIST

An online database of child and adolescent therapists around the U.S.

PSYCHOLOGY TODAY RESOURCES TO FIND AN ADULT THERAPIST

An online database of adult therapists around the U.S.

GOOD THERAPY RESOURCES TO FIND A CHILD THERAPIST

This website provides resources about child therapy, including an online database of therapists around the U.S.

GOOD THERAPY RESOURCES TO FIND AN ADULT THERAPIST

This website provides resources about adult therapy, including an online database of therapists around the U.S.

IF YOU OR YOUR CHILD ARE IN CRISIS OR FEELING SUICIDAL

Call National Suicide Prevention Lifeline **1-800-273-8255**
National Suicide Prevention Lifeline Chat
Crisis Text Line Text **HOME** to **74174**
Lifeline Crisis Chat: chat with crisis centers around the U.S.

IF YOU DO NOT THINK YOU CAN KEEP YOURSELF OR YOUR CHILD SAFE CALL EMERGENCY SERVICES (911) OR GO TO THE NEAREST HOSPITAL EMERGENCY ROOM/PSYCHIATRIC CRISIS CENTER
