# Supplementary material for: Empowering Anxious Parents to Manage Child Avoidance Behaviors: Randomized Control Trial of a Single-Session Intervention for Parental Accommodation
Source: JMIR Ment Health. 2021 Jul 6;8(7):e29538. doi: 10.2196/29538 (PMC8292931; doi:10.2196/29538)
Supplement: Multimedia Appendix 2 [file mental_v8i7e29538_app2.pdf]

CONSORT-EHEALTH (V 1.6.1) - Submission/Publication Form

The CONSORT-EHEALTH checklist is intended for authors of randomized trials evaluating web-based and Internet-based applications/interventions, including mobile interventions, electronic games (incl multiplayer games), social media, certain telehealth applications, and other interactive and/or networked electronic applications. Some of the items (e.g. all subitems under item 5 - description of the intervention) may also be applicable for other study designs.

The goal of the CONSORT EHEALTH checklist and guideline is to be
a) a guide for reporting for authors of RCTs,
b) to form a basis for appraisal of an ehealth trial (in terms of validity)

CONSORT-EHEALTH items/subitems are MANDATORY reporting items for studies published in the Journal of Medical Internet Research and other journals / scientific societies endorsing the checklist.

Items numbered 1., 2., 3., 4a., 4b etc are original CONSORT or CONSORT-NPT (non-pharmacologic treatment) items.
Items with Roman numerals (i., ii, iii, iv etc.) are CONSORT-EHEALTH extensions/clarifications.

As the CONSORT-EHEALTH checklist is still considered in a formative stage, we would ask that you also RATE ON A SCALE OF 1-5 how important/useful you feel each item is FOR THE PURPOSE OF THE CHECKLIST and reporting guideline (optional).

Mandatory reporting items are marked with a red *.
In the textboxes, either copy & paste the relevant sections from your manuscript into this form - please include any quotes from your manuscript in QUOTATION MARKS,
or answer directly by providing additional information not in the manuscript, or elaborating on why the item was not relevant for this study.

YOUR ANSWERS WILL BE PUBLISHED AS A SUPPLEMENTARY FILE TO YOUR PUBLICATION IN JMIR AND ARE CONSIDERED PART OF YOUR PUBLICATION (IF ACCEPTED).
Please fill in these questions diligently. Information will not be copyedited, so please use proper spelling and grammar, use correct capitalization, and avoid abbreviations.

DO NOT FORGET TO SAVE AS PDF _AND_ CLICK THE SUBMIT BUTTON SO YOUR ANSWERS ARE IN OUR DATABASE !!!

Citation Suggestion (if you append the pdf as Appendix we suggest to cite this paper in the caption):
Eysenbach G, CONSORT-EHEALTH Group
CONSORT-EHEALTH: Improving and Standardizing Evaluation Reports of Web-based and Mobile Health Interventions
J Med Internet Res 2011;13(4):e126
URL: http://www.jmir.org/2011/4/e126/
doi: 10.2196/jmir.1923
PMID: 22209829

* Required

Your name *

First Last

Jenna Sung

Primary Affiliation (short), City, Country *

University of Toronto, Toronto, Canada

Stony Brook University, Stony Brook, USA

Your e-mail address *

abc@gmail.com

Jenna.sung@stonybrook.edu

Title of your manuscript *

Provide the (draft) title of your manuscript.

Empowering Anxious Parents to Manage Child Avoidance Behaviors: Randomized Control Trial of a Single-Session Intervention for Parent Accommodation

Name of your App/Software/Intervention *

If there is a short and a long/alternate name, write the short name first and add the long name in brackets.

Project EMPOWER

Evaluated Version (if any)

e.g. "V1", "Release 2017-03-01", "Version 2.0.27913"

Not Applicable

Language(s) *

What language is the intervention/app in? If multiple languages are available, separate by comma (e.g. "English, French")

English

URL of your Intervention Website or App

e.g. a direct link to the mobile app on app in appstore (itunes, Google Play), or URL of the website. If the intervention is a DVD or hardware, you can also link to an Amazon page.

http://www.schleiderlab.org/empower.html

URL of an image/screenshot (optional)

https://drive.google.com/file/d/1vaqT05lDxOrJ8qa60bNhRO-Di8zlXOdj/view?usp=sharing

Accessibility *

Can an enduser access the intervention presently?

- (●) access is free and open
- ( ) access only for special usergroups, not open
- ( ) access is open to everyone, but requires payment/subscription/in-app purchases
- ( ) app/intervention no longer accessible
- ( ) Other:

Primary Medical Indication/Disease/Condition *

e.g. "Stress", "Diabetes", or define the target group in brackets after the condition, e.g. "Autism (Parents of children with)", "Alzheimers (Informal Caregivers of)"

Anxiety (Parents of children with)

Primary Outcomes measured in trial *

comma-separated list of primary outcomes reported in the trial

Family Accommodation, Distress Tolerance

Secondary/other outcomes

Are there any other outcomes the intervention is expected to affect?

Preparedness to manage child distress

Recommended "Dose" *

What do the instructions for users say on how often the app should be used?

- ○ Approximately Daily
- ○ Approximately Weekly
- ○ Approximately Monthly
- ○ Approximately Yearly
- ○ "as needed"
- ● Other: Single dose

Approx. Percentage of Users (starters) still using the app as recommended after 3 months *

- (selected) unknown / not evaluated
- 0-10%
- 11-20%
- 21-30%
- 31-40%
- 41-50%
- 51-60%
- 61-70%
- 71%-80%
- 81-90%
- 91-100%
- Other:

Overall, was the app/intervention effective? *

- (selected) yes: all primary outcomes were significantly better in intervention group vs control
- partly: SOME primary outcomes were significantly better in intervention group vs control
- no statistically significant difference between control and intervention
- potentially harmful: control was significantly better than intervention in one or more outcomes
- inconclusive: more research is needed
- Other:

Article Preparation Status/Stage *

At which stage in your article preparation are you currently (at the time you fill in this form)

- ( ) not submitted yet - in early draft status
- ( ) not submitted yet - in late draft status, just before submission
- ( ) submitted to a journal but not reviewed yet
- ( ) submitted to a journal and after receiving initial reviewer comments
- (•) submitted to a journal and accepted, but not published yet
- ( ) published
- ( ) Other:

Journal *

If you already know where you will submit this paper (or if it is already submitted), please provide the journal name (if it is not JMIR, provide the journal name under "other")

- ( ) not submitted yet / unclear where I will submit this
- ( ) Journal of Medical Internet Research (JMIR)
- ( ) JMIR mHealth and UHealth
- ( ) JMIR Serious Games
- (•) JMIR Mental Health
- ( ) JMIR Public Health
- ( ) JMIR Formative Research
- ( ) Other JMIR sister journal
- ( ) Other:

Is this a full powered effectiveness trial or a pilot/feasibility trial? *

- ( ) Pilot/feasibility
- (•) Fully powered

Manuscript tracking number *

If this is a JMIR submission, please provide the manuscript tracking number under "other" (The ms tracking number can be found in the submission acknowledgement email, or when you login as author in JMIR. If the paper is already published in JMIR, then the ms tracking number is the four-digit number at the end of the DOI, to be found at the bottom of each published article in JMIR)

- ○ no ms number (yet) / not (yet) submitted to / published in JMIR
- ◉ Other: 29538

TITLE AND ABSTRACT

1a) TITLE: Identification as a randomized trial in the title

1a) Does your paper address CONSORT item 1a? *

I.e does the title contain the phrase "Randomized Controlled Trial"? (if not, explain the reason under "other")

- ◉ yes
- ○ Other:

1a-i) Identify the mode of delivery in the title

Identify the mode of delivery. Preferably use “web-based” and/or “mobile” and/or “electronic game” in the title. Avoid ambiguous terms like “online”, “virtual”, “interactive”. Use “Internet-based” only if Intervention includes non-web-based Internet components (e.g. email), use “computer-based” or “electronic” only if offline products are used. Use “virtual” only in the context of “virtual reality” (3-D worlds). Use “online” only in the context of “online support groups”. Complement or substitute product names with broader terms for the class of products (such as “mobile” or “smart phone” instead of “iphone”), especially if the application runs on different platforms.

	1	2	3	4	5	
subitem not at all important	○	○	○	○	○	essential

Does your paper address subitem 1a-i? *

Copy and paste relevant sections from manuscript title (include quotes in quotation marks "like this" to indicate direct quotes from your manuscript), or elaborate on this item by providing additional information not in the ms, or briefly explain why the item is not applicable/relevant for your study

The mode of delivery is clearly addressed in the abstract, "Project EMPOWER: a web-based, self-guided SSI designed to reduce parent accommodation", and in the key words "Digital Mental Health"

1a-ii) Non-web-based components or important co-interventions in title

Mention non-web-based components or important co-interventions in title, if any (e.g., “with telephone support”).

	1	2	3	4	5	
subitem not at all important	○	○	○	○	○	essential

Does your paper address subitem 1a-ii?

Copy and paste relevant sections from manuscript title (include quotes in quotation marks "like this" to indicate direct quotes from your manuscript), or elaborate on this item by providing additional information not in the ms, or briefly explain why the item is not applicable/relevant for your study

This item is not applicable for the current study as non-web based components are not utilized.

1a-iii) Primary condition or target group in the title

Mention primary condition or target group in the title, if any (e.g., “for children with Type I Diabetes”)
Example: A Web-based and Mobile Intervention with Telephone Support for Children with Type I Diabetes: Randomized Controlled Trial

	1	2	3	4	5	
subitem not at all important	○	○	○	○	○	essential

Does your paper address subitem 1a-iii? *

Copy and paste relevant sections from manuscript title (include quotes in quotation marks "like this" to indicate direct quotes from your manuscript), or elaborate on this item by providing additional information not in the ms, or briefly explain why the item is not applicable/relevant for your study

"Empowering Anxious Parents to Manage Child Avoidance Behaviors: Randomized Control Trial of a Single-Session Intervention for Parent Accommodation"

1b) ABSTRACT: Structured summary of trial design, methods, results, and conclusions

NPT extension: Description of experimental treatment, comparator, care providers, centers, and blinding status.

1b-i) Key features/functionalities/components of the intervention and comparator in the METHODS section of the ABSTRACT

Mention key features/functionalities/components of the intervention and comparator in the abstract. If possible, also mention theories and principles used for designing the site. Keep in mind the needs of systematic reviewers and indexers by including important synonyms. (Note: Only report in the abstract what the main paper is reporting. If this information is missing from the main body of text, consider adding it)

	1	2	3	4	5	
subitem not at all important	○	○	○	○	○	essential

Does your paper address subitem 1b-i? *

Copy and paste relevant sections from the manuscript abstract (include quotes in quotation marks "like this" to indicate direct quotes from your manuscript), or elaborate on this item by providing additional information not in the ms, or briefly explain why the item is not applicable/relevant for your study

"301 parents reporting elevated anxiety symptoms with children ages 4-10 received either Project EMPOWER or an informational control (containing psychoeducational materials and resources)"

1b-ii) Level of human involvement in the METHODS section of the ABSTRACT

Clarify the level of human involvement in the abstract, e.g., use phrases like “fully automated” vs. “therapist/nurse/care provider/physician-assisted” (mention number and expertise of providers involved, if any). (Note: Only report in the abstract what the main paper is reporting. If this information is missing from the main body of text, consider adding it)

	1	2	3	4	5	
subitem not at all important	○	○	○	○	○	essential

Does your paper address subitem 1b-ii?

Copy and paste relevant sections from the manuscript abstract (include quotes in quotation marks "like this" to indicate direct quotes from your manuscript), or elaborate on this item by providing additional information not in the ms, or briefly explain why the item is not applicable/relevant for your study

The paper addresses the level of human involvement in the objectives section of the abstract: "This trial evaluated the acceptability and proximal effects of Project EMPOWER: a web-based, self-guided SSI designed to reduce parent accommodation, a parenting behavior known to increase anxiety risk in offspring"

1b-iii) Open vs. closed, web-based (self-assessment) vs. face-to-face assessments in the METHODS section of the ABSTRACT

Mention how participants were recruited (online vs. offline), e.g., from an open access website or from a clinic or a closed online user group (closed usergroup trial), and clarify if this was a purely web-based trial, or there were face-to-face components (as part of the intervention or for assessment). Clearly say if outcomes were self-assessed through questionnaires (as common in web-based trials). Note: In traditional offline trials, an open trial (open-label trial) is a type of clinical trial in which both the researchers and participants know which treatment is being administered. To avoid confusion, use “blinded” or “unblinded” to indicated the level of blinding instead of “open”, as “open” in web-based trials usually refers to “open access” (i.e. participants can self-enrol). (Note: Only report in the abstract what the main paper is reporting. If this information is missing from the main body of text, consider adding it)

	1	2	3	4	5	
subitem not at all important	○	○	○	○	○	essential

Does your paper address subitem 1b-iii?

Copy and paste relevant sections from the manuscript abstract (include quotes in quotation marks "like this" to indicate direct quotes from your manuscript), or elaborate on this item by providing additional information not in the ms, or briefly explain why the item is not applicable/relevant for your study

"301 parents reporting elevated anxiety symptoms with children ages 4-10 were recruited online to receive either Project EMPOWER or an informational control (containing psychoeducational materials and resources); parents self-reported their accommodation of child anxiety and overall distress tolerance at baseline and 2-week follow-up"

1b-iv) RESULTS section in abstract must contain use data

Report number of participants enrolled/assessed in each group, the use/uptake of the intervention (e.g., attrition/adherence metrics, use over time, number of logins etc.), in addition to primary/secondary outcomes. (Note: Only report in the abstract what the main paper is reporting. If this information is missing from the main body of text, consider adding it)

	1	2	3	4	5	
subitem not at all important	○	○	○	○	○	essential

Does your paper address subitem 1b-iv?

Copy and paste relevant sections from the manuscript abstract (include quotes in quotation marks "like this" to indicate direct quotes from your manuscript), or elaborate on this item by providing additional information not in the ms, or briefly explain why the item is not applicable/relevant for your study

The results section of the abstract report the primary/secondary outcomes: "Relative to control-group parents, parents who received Project EMPOWER reported significant reductions in their accommodation of child anxiety (d_s=0.61, P<.001), as well as significant increases in their distress tolerance (d_s=0.43, P<.001), from baseline to 2-week follow-up. Additionally, parents rated Project EMPOWER as highly acceptable (e.g., easy to use, helpful, engaging) per pre-registered benchmarks."

1b-v) CONCLUSIONS/DISCUSSION in abstract for negative trials

Conclusions/Discussions in abstract for negative trials: Discuss the primary outcome - if the trial is negative (primary outcome not changed), and the intervention was not used, discuss whether negative results are attributable to lack of uptake and discuss reasons. (Note: Only report in the abstract what the main paper is reporting. If this information is missing from the main body of text, consider adding it)

	1	2	3	4	5	
subitem not at all important	○	○	○	○	○	essential

Does your paper address subitem 1b-v?

Copy and paste relevant sections from the manuscript abstract (include quotes in quotation marks "like this" to indicate direct quotes from your manuscript), or elaborate on this item by providing additional information not in the ms, or briefly explain why the item is not applicable/relevant for your study

This subitem is not applicable

INTRODUCTION

2a) In INTRODUCTION: Scientific background and explanation of rationale

2a-i) Problem and the type of system/solution

Describe the problem and the type of system/solution that is object of the study: intended as stand-alone intervention vs. incorporated in broader health care program? Intended for a particular patient population? Goals of the intervention, e.g., being more cost-effective to other interventions, replace or complement other solutions? (Note: Details about the intervention are provided in “Methods” under 5)

	1	2	3	4	5	
subitem not at all important	○	○	○	○	○	essential

Does your paper address subitem 2a-i? *

Copy and paste relevant sections from the manuscript (include quotes in quotation marks "like this" to indicate direct quotes from your manuscript), or elaborate on this item by providing additional information not in the ms, or briefly explain why the item is not applicable/relevant for your study

"Anxiety disorders are among the most common, debilitating forms of childhood psychopathology, affecting 8.3-27.0% of youth before age 18 [1,2]. Child anxiety increases risk for psychiatric comorbidities across the lifespan [3], creates significant burdens for caregivers [4], and carries stark societal costs [5,6]. Although numerous interventions have been developed to treat youth anxiety disorders, up to 82.2% of US youth with anxiety will not receive adequate care [7]. Several factors may explain this discrepancy, including the length and cost of existing treatments and limited accessibility for families in need. Together, these factors create a pressing need for accessible, brief preventive programs to decrease odds of anxiety disorder onset in at-risk youth."

"Thus, well-targeted SSIs may offer cost-effective additions or alternatives to traditional care for anxiety in youth.."

2a-ii) Scientific background, rationale: What is known about the (type of) system

Scientific background, rationale: What is known about the (type of) system that is the object of the study (be sure to discuss the use of similar systems for other conditions/diagnoses, if appropiate), motivation for the study, i.e. what are the reasons for and what is the context for this specific study, from which stakeholder viewpoint is the study performed, potential impact of findings [2]. Briefly justify the choice of the comparator.

	1	2	3	4	5	
subitem not at all important	○	○	○	○	○	essential

Does your paper address subitem 2a-ii? *

Copy and paste relevant sections from the manuscript (include quotes in quotation marks "like this" to indicate direct quotes from your manuscript), or elaborate on this item by providing additional information not in the ms, or briefly explain why the item is not applicable/relevant for your study

"However, most existing SSIs for child anxiety target populations already experiencing clinical distress, leaving a need for options that may prevent anxiety in vulnerable children. Given that family factors play a crucial role in the etiology of child anxiety [1], SSIs targeting parents and their interactions with offspring may be a promising approach to preventing youth anxiety [9]. Thus, this trial examined the acceptability and short-term effects of a novel, web-based, self-guided SSI targeting parental accommodation: a well-established, potentially modifiable risk factor for child anxiety [10–13]."

2b) In INTRODUCTION: Specific objectives or hypotheses

Does your paper address CONSORT subitem 2b? *

Copy and paste relevant sections from the manuscript (include quotes in quotation marks "like this" to indicate direct quotes from your manuscript), or elaborate on this item by providing additional information not in the ms, or briefly explain why the item is not applicable/relevant for your study

"Thus, this trial examined the acceptability and short-term effects of a novel, web-based, self-guided SSI targeting parental accommodation: a well-established, potentially modifiable risk factor for child anxiety [10–13]."

"We predicted that parents would report larger declines in self-reported accommodation behaviors (primary outcome) and larger increases in distress tolerance (secondary outcome) in the ORR+EMPOWER group, relative to the ORR+waitlist, from baseline to 2-week follow-up. We also predicted that parents completing Project EMPOWER would subjectively perceive larger pre-to immediate-post-SSI increases in their ability to help their child manage distressing situations, relative to control-group parents. Finally, we predicted that parents completing Project EMPOWER would rate it as acceptable (enjoyable, worth recommending to other parents, and personally helpful). "

METHODS

3a) Description of trial design (such as parallel, factorial) including allocation ratio

Does your paper address CONSORT subitem 3a? *

Copy and paste relevant sections from the manuscript (include quotes in quotation marks "like this" to indicate direct quotes from your manuscript), or elaborate on this item by providing additional information not in the ms, or briefly explain why the item is not applicable/relevant for your study

"After clicking on a social media-based advertisement, parents were directed to an informational study webpage inviting them to complete an online eligibility screener. Eligible parents then reviewed an online consent form inviting them to participate. Parents could initiate the study at any time and location, using any internet-equipped device (smartphone, laptop, tablet). After starting the study, participants first completed pre-intervention self-report questionnaires, detailed below. Within the same survey, participants were randomized via Qualtrics (1:1 allocation ratio) to either receive Online Resources and Referrals (ORR) and immediate access to Project EMPOWER (intervention condition), or ORR and delayed Project EMPOWER access after the two-week follow-up (control condition). Those in the intervention condition also completed the Program Feedback Scale, along with other post-intervention surveys, immediately following Project EMPOWER completion. Two weeks later, all parents—regardless of condition—were invited to complete follow up questionnaires. Parents in the control condition were then invited to complete Project EMPOWER, if they were interested in doing so (completion of Project EMPOWER subsequent to follow-up questionnaires was optional, not part of the study). Thus, all participants were able to complete Project EMPOWERs, either immediately or after a 2-week delay."

3b) Important changes to methods after trial commencement (such as eligibility criteria), with reasons

Does your paper address CONSORT subitem 3b? *

Copy and paste relevant sections from the manuscript (include quotes in quotation marks "like this" to indicate direct quotes from your manuscript), or elaborate on this item by providing additional information not in the ms, or briefly explain why the item is not applicable/relevant for your study

This subitem is not applicable

3b-i) Bug fixes, Downtimes, Content Changes

Bug fixes, Downtimes, Content Changes: ehealth systems are often dynamic systems. A description of changes to methods therefore also includes important changes made on the intervention or comparator during the trial (e.g., major bug fixes or changes in the functionality or content) (5-iii) and other “unexpected events” that may have influenced study design such as staff changes, system failures/downtimes, etc. [2].

	1	2	3	4	5	
subitem not at all important	○	○	○	○	○	essential

Does your paper address subitem 3b-i?

Copy and paste relevant sections from the manuscript (include quotes in quotation marks "like this" to indicate direct quotes from your manuscript), or elaborate on this item by providing additional information not in the ms, or briefly explain why the item is not applicable/relevant for your study

This subitem is not applicable

4a) Eligibility criteria for participants

Does your paper address CONSORT subitem 4a? *

Copy and paste relevant sections from the manuscript (include quotes in quotation marks "like this" to indicate direct quotes from your manuscript), or elaborate on this item by providing additional information not in the ms, or briefly explain why the item is not applicable/relevant for your study

"Participants were eligible for the study if they (1) reported subclinical or greater anxiety symptoms (a score of >40 on the Penn State Worry Questionnaire—PSWQ), because children with higher-anxiety parents are at elevated risk for developing anxiety themselves, and higher-anxiety parents report engaging in more accommodation than do lower-anxiety parents [17]; (2) had at least one child between 4-10 years old; and (3) endorsed comfort with English (intervention materials were available in English only). This specific child age-range was selected because it encompasses the age of onset for common child anxiety disorders [23]; it also matches the age-range for which parent-focused interventions are often designed [24]."

4a-i) Computer / Internet literacy

Computer / Internet literacy is often an implicit “de facto” eligibility criterion - this should be explicitly clarified.

	1	2	3	4	5	
subitem not at all important	○	○	○	○	○	essential

Does your paper address subitem 4a-i?

Copy and paste relevant sections from the manuscript (include quotes in quotation marks "like this" to indicate direct quotes from your manuscript), or elaborate on this item by providing additional information not in the ms, or briefly explain why the item is not applicable/relevant for your study

Your answer

4a-ii) Open vs. closed, web-based vs. face-to-face assessments:

Open vs. closed, web-based vs. face-to-face assessments: Mention how participants were recruited (online vs. offline), e.g., from an open access website or from a clinic, and clarify if this was a purely web-based trial, or there were face-to-face components (as part of the intervention or for assessment), i.e., to what degree got the study team to know the participant. In online-only trials, clarify if participants were quasi-anonymous and whether having multiple identities was possible or whether technical or logistical measures (e.g., cookies, email confirmation, phone calls) were used to detect/prevent these.

	1	2	3	4	5	
subitem not at all important	○	○	○	○	○	essential

Does your paper address subitem 4a-ii? *

Copy and paste relevant sections from the manuscript (include quotes in quotation marks "like this" to indicate direct quotes from your manuscript), or elaborate on this item by providing additional information not in the ms, or briefly explain why the item is not applicable/relevant for your study

"After clicking on a social media-based advertisement, parents were directed to an informational study webpage inviting them to complete an online eligibility screener. Eligible parents then reviewed an online consent form inviting them to participate. Parents could initiate the study at any time and location, using any internet-equipped device (smartphone, laptop, tablet). After starting the study, participants first completed pre-intervention self-report questionnaires, detailed below. Within the same survey, participants were randomized via Qualtrics (1:1 allocation ratio) to either receive Online Resources and Referrals (ORR) and immediate access to Project EMPOWER (intervention condition), or ORR and delayed Project EMPOWER access after the two-week follow-up (control condition). Those in the intervention condition also completed the Program Feedback Scale, along with other post-intervention surveys, immediately following Project EMPOWER completion. Two weeks later, all parents—regardless of condition—were invited to complete follow up questionnaires. Parents in the control condition were then invited to complete Project EMPOWER, if they were interested in doing so (completion of Project EMPOWER subsequent to follow-up questionnaires was optional, not part of the study). Thus, all participants were able to complete Project EMPOWERs, either immediately or after a 2-week delay."

"Project EMPOWER (freely available for anonymous completion at www.schleiderlab.org/EMPOWER) is a web-based, self-guided SSI for parents that takes 20-30 minutes to complete."

4a-iii) Information giving during recruitment

Information given during recruitment. Specify how participants were briefed for recruitment and in the informed consent procedures (e.g., publish the informed consent documentation as appendix, see also item X26), as this information may have an effect on user self-selection, user expectation and may also bias results.

	1	2	3	4	5	
subitem not at all important	○	○	○	○	○	essential

Does your paper address subitem 4a-iii?

Copy and paste relevant sections from the manuscript (include quotes in quotation marks "like this" to indicate direct quotes from your manuscript), or elaborate on this item by providing additional information not in the ms, or briefly explain why the item is not applicable/relevant for your study

"After clicking on a social media-based advertisement, parents were directed to an informational study webpage inviting them to complete an online eligibility screener. Eligible parents then reviewed an online consent form inviting them to participate"

4b) Settings and locations where the data were collected

Does your paper address CONSORT subitem 4b? *

Copy and paste relevant sections from the manuscript (include quotes in quotation marks "like this" to indicate direct quotes from your manuscript), or elaborate on this item by providing additional information not in the ms, or briefly explain why the item is not applicable/relevant for your study

"Parents could initiate the study at any time and location, using any internet-equipped device (smartphone, laptop, tablet)."

4b-i) Report if outcomes were (self-)assessed through online questionnaires

Clearly report if outcomes were (self-)assessed through online questionnaires (as common in web-based trials) or otherwise.

	1	2	3	4	5	
subitem not at all important	○	○	○	○	○	essential

Does your paper address subitem 4b-i? *

Copy and paste relevant sections from the manuscript (include quotes in quotation marks "like this" to indicate direct quotes from your manuscript), or elaborate on this item by providing additional information not in the ms, or briefly explain why the item is not applicable/relevant for your study

"After starting the study, participants first completed pre-intervention self-report questionnaires, detailed below"

4b-ii) Report how institutional affiliations are displayed

Report how institutional affiliations are displayed to potential participants [on ehealth media], as affiliations with prestigious hospitals or universities may affect volunteer rates, use, and reactions with regards to an intervention.(Not a required item – describe only if this may bias results)

	1	2	3	4	5	
subitem not at all important	○	○	○	○	○	essential

Does your paper address subitem 4b-ii?

Copy and paste relevant sections from the manuscript (include quotes in quotation marks "like this" to indicate direct quotes from your manuscript), or elaborate on this item by providing additional information not in the ms, or briefly explain why the item is not applicable/relevant for your study

Your answer

5) The interventions for each group with sufficient details to allow replication, including how and when they were actually administered

5-i) Mention names, credential, affiliations of the developers, sponsors, and owners

Mention names, credential, affiliations of the developers, sponsors, and owners [6] (if authors/evaluators are owners or developer of the software, this needs to be declared in a “Conflict of interest” section or mentioned elsewhere in the manuscript).

	1	2	3	4	5	
subitem not at all important	○	○	○	○	○	essential

Does your paper address subitem 5-i?

Copy and paste relevant sections from the manuscript (include quotes in quotation marks "like this" to indicate direct quotes from your manuscript), or elaborate on this item by providing additional information not in the ms, or briefly explain why the item is not applicable/relevant for your study

"This project was funded by the Psi Chi Honor Society’s Graduate Research Grant. JYS receives funding from the Health Policy Research Scholars Program. JLS receives research support from the National Institutes of Health (DP5OD28123), the Klingenstein Third Generation Foundation, the American Psychological Foundation, the Upswing Fund, and Limbix, Inc. JLS is also a co-author of a therapeutic workbook for youth (published by New Harbinger Publications) and co-editing a book on low-intensity mental health interventions for youth with Oxford University Press. The authors report no other financial conflicts"

5-ii) Describe the history/development process

Describe the history/development process of the application and previous formative evaluations (e.g., focus groups, usability testing), as these will have an impact on adoption/use rates and help with interpreting results.

	1	2	3	4	5	
subitem not at all important	○	○	○	○	○	essential

Does your paper address subitem 5-ii?

Copy and paste relevant sections from the manuscript (include quotes in quotation marks "like this" to indicate direct quotes from your manuscript), or elaborate on this item by providing additional information not in the ms, or briefly explain why the item is not applicable/relevant for your study

Your answer

5-iii) Revisions and updating

Revisions and updating. Clearly mention the date and/or version number of the application/intervention (and comparator, if applicable) evaluated, or describe whether the intervention underwent major changes during the evaluation process, or whether the development and/or content was “frozen” during the trial. Describe dynamic components such as news feeds or changing content which may have an impact on the replicability of the intervention (for unexpected events see item 3b).

	1	2	3	4	5	
subitem not at all important	○	○	○	○	○	essential

Does your paper address subitem 5-iii?

Copy and paste relevant sections from the manuscript (include quotes in quotation marks "like this" to indicate direct quotes from your manuscript), or elaborate on this item by providing additional information not in the ms, or briefly explain why the item is not applicable/relevant for your study

Your answer

5-iv) Quality assurance methods

Provide information on quality assurance methods to ensure accuracy and quality of information provided [1], if applicable.

	1	2	3	4	5	
subitem not at all important	○	○	○	○	○	essential

Does your paper address subitem 5-iv?

Copy and paste relevant sections from the manuscript (include quotes in quotation marks "like this" to indicate direct quotes from your manuscript), or elaborate on this item by providing additional information not in the ms, or briefly explain why the item is not applicable/relevant for your study

Your answer

5-v) Ensure replicability by publishing the source code, and/or providing screenshots/screen-capture video, and/or providing flowcharts of the algorithms used

Ensure replicability by publishing the source code, and/or providing screenshots/screen-capture video, and/or providing flowcharts of the algorithms used. Replicability (i.e., other researchers should in principle be able to replicate the study) is a hallmark of scientific reporting.

	1	2	3	4	5	
subitem not at all important	○	○	○	○	○	essential

Does your paper address subitem 5-v?

Copy and paste relevant sections from the manuscript (include quotes in quotation marks "like this" to indicate direct quotes from your manuscript), or elaborate on this item by providing additional information not in the ms, or briefly explain why the item is not applicable/relevant for your study

Your answer

5-vi) Digital preservation

Digital preservation: Provide the URL of the application, but as the intervention is likely to change or disappear over the course of the years; also make sure the intervention is archived (Internet Archive, webcitation.org, and/or publishing the source code or screenshots/videos alongside the article). As pages behind login screens cannot be archived, consider creating demo pages which are accessible without login.

	1	2	3	4	5	
subitem not at all important	○	○	○	○	○	essential

Does your paper address subitem 5-vi?

Copy and paste relevant sections from the manuscript (include quotes in quotation marks "like this" to indicate direct quotes from your manuscript), or elaborate on this item by providing additional information not in the ms, or briefly explain why the item is not applicable/relevant for your study

Your answer

5-vii) Access

Access: Describe how participants accessed the application, in what setting/context, if they had to pay (or were paid) or not, whether they had to be a member of specific group. If known, describe how participants obtained “access to the platform and Internet” [1]. To ensure access for editors/reviewers/readers, consider to provide a “backdoor” login account or demo mode for reviewers/readers to explore the application (also important for archiving purposes, see vi).

	1	2	3	4	5	
subitem not at all important	○	○	○	○	○	essential

Does your paper address subitem 5-vii? *

Copy and paste relevant sections from the manuscript (include quotes in quotation marks "like this" to indicate direct quotes from your manuscript), or elaborate on this item by providing additional information not in the ms, or briefly explain why the item is not applicable/relevant for your study

"Project EMPOWER (freely available for anonymous completion on the project’s website [36]) is a web-based, self-guided SSI for parents that takes 20-30 minutes to complete"

"After clicking on a social media advertisement, parents were directed to an informational study webpage that invited them to complete a web-based eligibility screener. Eligible parents then reviewed a web-based consent form that invited them to participate. Parents could initiate the study at any time and location, using any internet-equipped device (smartphone, laptop, or tablet device)."

5-viii) Mode of delivery, features/functionalities/components of the intervention and comparator, and the theoretical framework

Describe mode of delivery, features/functionalities/components of the intervention and comparator, and the theoretical framework [6] used to design them (instructional strategy [1], behaviour change techniques, persuasive features, etc., see e.g., [7, 8] for terminology). This includes an in-depth description of the content (including where it is coming from and who developed it) [1]," whether [and how] it is tailored to individual circumstances and allows users to track their progress and receive feedback" [6]. This also includes a description of communication delivery channels and – if computer-mediated communication is a component – whether communication was synchronous or asynchronous [6]. It also includes information on presentation strategies [1], including page design principles, average amount of text on pages, presence of hyperlinks to other resources, etc. [1].

	1	2	3	4	5	
subitem not at all important	○	○	○	○	○	essential

Does your paper address subitem 5-viii? *

Copy and paste relevant sections from the manuscript (include quotes in quotation marks "like this" to indicate direct quotes from your manuscript), or elaborate on this item by providing additional information not in the ms, or briefly explain why the item is not applicable/relevant for your study

"Project EMPOWER (freely available for anonymous completion on the project's website [36]) is a web-based, self-guided SSI for parents that takes 20-30 minutes to complete. The program includes 5 main elements, which are based on current recommended practices in SSI design [28] and existing, therapist-delivered interventions targeting parental accommodation [15,21]:
1. Psychoeducation on child anxiety and avoidance, along with how parental accommodation can inadvertently foster child anxiety;
2. Information on how parents can better identify children's patterns of avoidance and encourage "brave behavior," instead;
3. An exercise that guides parents in creating a personalized, step-by-step "action plan" for promoting brave, approach-oriented behaviors (rather than anxiety-driven avoidance) in their own child;
4. A segment intended to normalize parent distress responses in response to anxiety in offspring, including a rationale for why encouraging "brave behaviors"—despite being emotionally challenging for caregivers—ultimately bolsters children's well-being and resilience; and
5. A vignette exercise in which parents read about another family's difficulty managing child anxiety; parents identify various elements of the "anxiety cycle" (in accordance with psychoeducation provided previously) and generate possible solutions for the parents described in the vignette, which are based on their newfound knowledge of promoting "brave behavior" in the youth."

"ORR included an information sheet containing a list of web-based psychoeducational resources (videos, books, web-based toolkits, etc) on anxiety, hotlines, and resources on finding mental health treatment around the United States. ORR did not include any psychoeducational components explicitly designed to reduce parental accommodation of child anxiety. For the full content of ORR is provided in Multimedia Appendix 1."

5-ix) Describe use parameters

Describe use parameters (e.g., intended “doses” and optimal timing for use). Clarify what instructions or recommendations were given to the user, e.g., regarding timing, frequency, heaviness of use, if any, or was the intervention used ad libitum.

	1	2	3	4	5	
subitem not at all important	○	○	○	○	○	essential

Does your paper address subitem 5-ix?

Copy and paste relevant sections from the manuscript (include quotes in quotation marks "like this" to indicate direct quotes from your manuscript), or elaborate on this item by providing additional information not in the ms, or briefly explain why the item is not applicable/relevant for your study

Your answer

5-x) Clarify the level of human involvement

Clarify the level of human involvement (care providers or health professionals, also technical assistance) in the e-intervention or as co-intervention (detail number and expertise of professionals involved, if any, as well as “type of assistance offered, the timing and frequency of the support, how it is initiated, and the medium by which the assistance is delivered”. It may be necessary to distinguish between the level of human involvement required for the trial, and the level of human involvement required for a routine application outside of a RCT setting (discuss under item 21 – generalizability).

	1	2	3	4	5	
subitem not at all important	○	○	○	○	○	essential

Does your paper address subitem 5-x?

Copy and paste relevant sections from the manuscript (include quotes in quotation marks "like this" to indicate direct quotes from your manuscript), or elaborate on this item by providing additional information not in the ms, or briefly explain why the item is not applicable/relevant for your study

Your answer

5-xi) Report any prompts/reminders used

Report any prompts/reminders used: Clarify if there were prompts (letters, emails, phone calls, SMS) to use the application, what triggered them, frequency etc. It may be necessary to distinguish between the level of prompts/reminders required for the trial, and the level of prompts/reminders for a routine application outside of a RCT setting (discuss under item 21 – generalizability).

	1	2	3	4	5	
subitem not at all important	○	○	○	○	○	essential

Does your paper address subitem 5-xi? *

Copy and paste relevant sections from the manuscript (include quotes in quotation marks "like this" to indicate direct quotes from your manuscript), or elaborate on this item by providing additional information not in the ms, or briefly explain why the item is not applicable/relevant for your study

"First, although the completely web-based study design allowed for a large sample size and rapid, low-cost recruitment through social media, the lack of monetary compensation likely contributed to substantial attrition at follow-up (61.13%), despite scheduled email reminders"

5-xii) Describe any co-interventions (incl. training/support)

Describe any co-interventions (incl. training/support): Clearly state any interventions that are provided in addition to the targeted eHealth intervention, as ehealth intervention may not be designed as stand-alone intervention. This includes training sessions and support [1]. It may be necessary to distinguish between the level of training required for the trial, and the level of training for a routine application outside of a RCT setting (discuss under item 21 – generalizability.

	1	2	3	4	5	
subitem not at all important	○	○	○	○	○	essential

Does your paper address subitem 5-xii? *

Copy and paste relevant sections from the manuscript (include quotes in quotation marks "like this" to indicate direct quotes from your manuscript), or elaborate on this item by providing additional information not in the ms, or briefly explain why the item is not applicable/relevant for your study

This subitem is not applicable as project EMPOWER does not have require any additional training/support

6a) Completely defined pre-specified primary and secondary outcome measures, including how and when they were assessed

Does your paper address CONSORT subitem 6a? *

Copy and paste relevant sections from the manuscript (include quotes in quotation marks "like this" to indicate direct quotes from your manuscript), or elaborate on this item by providing additional information not in the ms, or briefly explain why the item is not applicable/relevant for your study

"Parent Accommodation of Child Anxiety (Primary Outcome)
Using the Family Accommodation Scale—Anxiety (FASA) [10], parents rated agreement with 9-items, which reflected the extent to which they accommodate their child's anxiety symptoms or avoidance behaviors. Higher mean scores indicate more frequent parental accommodation. As a primary outcome measure, the FASA was administered at baseline and 2-week follow up to all participants. The FASA has demonstrated excellent psychometric properties across numerous studies [10]. Here we used α values of .87 and .85 at baseline and 2-week follow-up, respectively.

Parent Distress Tolerance (Secondary Outcome)
Using the 16-item Distress Tolerance Scale (DTS) [25]—a valid, reliable measure of overall distress tolerance in adults—parents rated their perceived ability to experience and withstand distressing emotional states on a 5-point scale. Higher mean DTS scores reflect lower levels of distress tolerance. As a secondary outcome measure, DTS was administered at baseline and 2-week follow to all study participants. Here we used α values of .86 and .88 at baseline and 2-week follow-up, respectively."

"Perceived Change in Preparedness to Help Children Manage Distress
A single-item measure that gauges participants' perceived changes in their ability to help their children manage distressing situations was adapted for this study [28]. All participants were asked to rate their agreement with a single-item statement on a 5-point scale, either immediately after completing project EMPOWER (intervention condition) or immediately after being presented with psychoeducational materials (informational waitlist condition): "Compared to before you started this survey, how prepared do you feel to help your child manage distressing situations?" This item was administered immediately post SSI only for the intervention group as a secondary, exploratory outcome.

Intervention Acceptability
Parents in the intervention condition completed the Program Feedback Scale (PFS) [29]—a reliable and valid measure routinely used to assess acceptability and user perceptions of web-based, self-guided SSIs. The PFS asks participants to rate 7 statements on a 5-point scale (scores ranging 1-5) and share what they liked and what they would change about the SSI, in an open-response format. A mean score of ≥3 indicates acceptability and positive program evaluation. The PFS was administered at post-SSI to parents assigned to the intervention condition to assess program acceptability."

6a-i) Online questionnaires: describe if they were validated for online use and apply CHERRIES items to describe how the questionnaires were designed/deployed

If outcomes were obtained through online questionnaires, describe if they were validated for online use and apply CHERRIES items to describe how the questionnaires were designed/deployed [9].

	1	2	3	4	5	
subitem not at all important	○	○	○	○	○	essential

Does your paper address subitem 6a-i?

Copy and paste relevant sections from manuscript text

Your answer

6a-ii) Describe whether and how "use" (including intensity of use/dosage) was defined/measured/monitored

Describe whether and how "use" (including intensity of use/dosage) was defined/measured/monitored (logins, logfile analysis, etc.). Use/adoption metrics are important process outcomes that should be reported in any ehealth trial.

	1	2	3	4	5	
subitem not at all important	○	○	○	○	○	essential

Does your paper address subitem 6a-ii?

Copy and paste relevant sections from manuscript text

Your answer

6a-iii) Describe whether, how, and when qualitative feedback from participants was obtained

Describe whether, how, and when qualitative feedback from participants was obtained (e.g., through emails, feedback forms, interviews, focus groups).

	1	2	3	4	5	
subitem not at all important	○	○	○	○	○	essential

Does your paper address subitem 6a-iii?

Copy and paste relevant sections from manuscript text

"Parents in the intervention condition completed the Program Feedback Scale (PFS) [29]—a reliable and valid measure routinely used to assess acceptability and user perceptions of web-based, self-guided SSIs. The PFS asks participants to rate 7 statements on a 5-point scale (scores ranging 1-5) and share what they liked and what they would change about the SSI, in an open-response format. A mean score of ≥3 indicates acceptability and positive program evaluation. The PFS was administered at post-SSI to parents assigned to the intervention condition to assess program acceptability."

6b) Any changes to trial outcomes after the trial commenced, with reasons

Does your paper address CONSORT subitem 6b? *

Copy and paste relevant sections from the manuscript (include quotes in quotation marks "like this" to indicate direct quotes from your manuscript), or elaborate on this item by providing additional information not in the ms, or briefly explain why the item is not applicable/relevant for your study

This subitem is not applicable

7a) How sample size was determined

NPT: When applicable, details of whether and how the clustering by care provides or centers was addressed

7a-i) Describe whether and how expected attrition was taken into account when calculating the sample size

Describe whether and how expected attrition was taken into account when calculating the sample size.

	1	2	3	4	5	
subitem not at all important	○	○	○	○	○	essential

Does your paper address subitem 7a-i?

Copy and paste relevant sections from manuscript title (include quotes in quotation marks "like this" to indicate direct quotes from your manuscript), or elaborate on this item by providing additional information not in the ms, or briefly explain why the item is not applicable/relevant for your study

"Using G*Power 3.1, sample sizes needed to detect group differences in the primary outcome (changes in accommodation from baseline to follow-up) between the intervention and control groups of small (.2), medium (.5), and large effects (.8) based on an F-test, linear multiple regression with α=.05, and power=0.80, were 395, 55, and 25, respectively. Thus, our sample (n=301) offered sufficient power to detect a small-to-medium between-groups effects (consistent with effect sizes observed in previous randomized trials on web-based SSIs) [30]."

7b) When applicable, explanation of any interim analyses and stopping guidelines

Does your paper address CONSORT subitem 7b? *

Copy and paste relevant sections from the manuscript (include quotes in quotation marks "like this" to indicate direct quotes from your manuscript), or elaborate on this item by providing additional information not in the ms, or briefly explain why the item is not applicable/relevant for your study

Interim analyses are not applicable to this study. The stopping guidelines were as follows: "Study recruitment began in July 2020 and ended in August 2020 once the target number of participants was achieved"

8a) Method used to generate the random allocation sequence

NPT: When applicable, how care providers were allocated to each trial group

Does your paper address CONSORT subitem 8a? *

Copy and paste relevant sections from the manuscript (include quotes in quotation marks "like this" to indicate direct quotes from your manuscript), or elaborate on this item by providing additional information not in the ms, or briefly explain why the item is not applicable/relevant for your study

"Within the same survey, participants were randomized via Qualtrics (1:1 allocation ratio) to receive either ORR and immediate access to project EMPOWER (intervention condition) or ORR and delayed access to project EMPOWER after the 2-week follow-up (control condition)"

8b) Type of randomisation; details of any restriction (such as blocking and block size)

Does your paper address CONSORT subitem 8b? *

Copy and paste relevant sections from the manuscript (include quotes in quotation marks "like this" to indicate direct quotes from your manuscript), or elaborate on this item by providing additional information not in the ms, or briefly explain why the item is not applicable/relevant for your study

Randomization did not include any restrictions for this study. Participants were randomized to two groups: "Within the same survey, participants were randomized via Qualtrics (1:1 allocation ratio) to receive either ORR and immediate access to project EMPOWER (intervention condition) or ORR and delayed access to project EMPOWER after the 2-week follow-up (control condition)"

9) Mechanism used to implement the random allocation sequence (such as sequentially numbered containers), describing any steps taken to conceal the sequence until interventions were assigned

Does your paper address CONSORT subitem 9? *

Copy and paste relevant sections from the manuscript (include quotes in quotation marks "like this" to indicate direct quotes from your manuscript), or elaborate on this item by providing additional information not in the ms, or briefly explain why the item is not applicable/relevant for your study

The mechanisms used by the Qualtrics survey is not known to the authors:
"Within the same survey, participants were randomized via Qualtrics (1:1 allocation ratio) to receive either ORR and immediate access to project EMPOWER (intervention condition) or ORR and delayed access to project EMPOWER after the 2-week follow-up (control condition)"

10) Who generated the random allocation sequence, who enrolled participants, and who assigned participants to interventions

Does your paper address CONSORT subitem 10? *

Copy and paste relevant sections from the manuscript (include quotes in quotation marks "like this" to indicate direct quotes from your manuscript), or elaborate on this item by providing additional information not in the ms, or briefly explain why the item is not applicable/relevant for your study

The entire study was automated via Qualtrics: "Within the same survey, participants were randomized via Qualtrics (1:1 allocation ratio) to receive either ORR and immediate access to project EMPOWER (intervention condition) or ORR and delayed access to project EMPOWER after the 2-week follow-up (control condition)"

11a) If done, who was blinded after assignment to interventions (for example, participants, care providers, those assessing outcomes) and how

NPT: Whether or not administering co-interventions were blinded to group assignment

11a-i) Specify who was blinded, and who wasn't

Specify who was blinded, and who wasn't. Usually, in web-based trials it is not possible to blind the participants [1, 3] (this should be clearly acknowledged), but it may be possible to blind outcome assessors, those doing data analysis or those administering co-interventions (if any).

	1	2	3	4	5	
subitem not at all important	○	○	○	○	○	essential

Does your paper address subitem 11a-i? *

Copy and paste relevant sections from the manuscript (include quotes in quotation marks "like this" to indicate direct quotes from your manuscript), or elaborate on this item by providing additional information not in the ms, or briefly explain why the item is not applicable/relevant for your study

This subitem is not applicable to this study as the data collection process was fully automated via Qualtrics

11a-ii) Discuss e.g., whether participants knew which intervention was the “intervention of interest” and which one was the “comparator”

Informed consent procedures (4a-ii) can create biases and certain expectations - discuss e.g., whether participants knew which intervention was the “intervention of interest” and which one was the “comparator”.

	1	2	3	4	5	
subitem not at all important	○	○	○	○	○	essential

Does your paper address subitem 11a-ii?

Copy and paste relevant sections from the manuscript (include quotes in quotation marks "like this" to indicate direct quotes from your manuscript), or elaborate on this item by providing additional information not in the ms, or briefly explain why the item is not applicable/relevant for your study

Your answer

11b) If relevant, description of the similarity of interventions

(this item is usually not relevant for ehealth trials as it refers to similarity of a placebo or sham intervention to a active medication/intervention)

Does your paper address CONSORT subitem 11b? *

Copy and paste relevant sections from the manuscript (include quotes in quotation marks "like this" to indicate direct quotes from your manuscript), or elaborate on this item by providing additional information not in the ms, or briefly explain why the item is not applicable/relevant for your study

"ORR did not include any psychoeducational components explicitly designed to reduce parental accommodation of child anxiety."

12a) Statistical methods used to compare groups for primary and secondary outcomes

NPT: When applicable, details of whether and how the clustering by care providers or centers was addressed

Does your paper address CONSORT subitem 12a? *

Copy and paste relevant sections from the manuscript (include quotes in quotation marks "like this" to indicate direct quotes from your manuscript), or elaborate on this item by providing additional information not in the ms, or briefly explain why the item is not applicable/relevant for your study

"Analysis Plan
The entire preregistration can be found on ClinicalTrials.gov (NCT04453865). Deidentified data and code for all preregistered analyses are available on the Open Science Framework [37].

Effects of Project EMPOWER on Primary and Secondary Outcomes
To assess the effects of the intervention on parent's accommodation levels and distress tolerance from baseline to 2-week follow up, we used a multiple linear regression approach with the intervention condition (1=ORR+EMPOWER; 0=ORR+waitlist), baseline accommodation levels, and parental distress as predictor variables to examine whether participants in each condition saw a differential reduction in outcome variables. Using the MOTE R Package, we also reported the Cohen d effect sizes and 95% Cis for within-group (d_av; reflecting intervention effects for changes before the intervention up to follow-up) and between-group (d_s; reflecting changes in outcome before the intervention up to follow-up in the 2 groups) differences in both accommodation and distress tolerance levels [31].
Perceived Change in Preparedness to Manage Child Anxiety Before and After the Survey
A 2-sample t test was performed to determine whether the overall, subjectively detectable pre-to-post changes in "preparedness to help their child manage anxiety" significantly differed between parents who completed project EMPOWER immediately, compared to control-group parents.

Intervention Acceptability
We examined overall and item-level mean PFS scores among parents who completed project EMPOWER. Mean and item-level scores of >3 or higher on any item (on a 5-point scale) reflected the endorsement of the program's acceptability (eg, positive feedback), either for that specific item or overall.

Project EMPOWER Completion Rates
Operational definitions of differential "program completer" status among parents assigned to the project EMPOWER condition were preregistered prior to data analysis. Full completers were those who reached the final page of the intervention, thus receiving the full "dose" of intended materials (approximate completion time: 25-30 minutes); personalized plan completers completed all psychoeducational content in project EMPOWER and finished their personalized plan for promoting brave behavior in their child (approximate completion time: 20-25 minutes); psychoeducational content completers completed all psychoeducational content, but not a personalized plan (approximate completion time: 10-15 minutes); and partial completers began the intervention but did not reach any of the above-mentioned program benchmarks. We report completion rates at each level, among parents assigned to the project EMPOWER condition, in the CONSORT diagram (Figure 1)."

12a-i) Imputation techniques to deal with attrition / missing values

Imputation techniques to deal with attrition / missing values: Not all participants will use the intervention/comparator as intended and attrition is typically high in ehealth trials. Specify how participants who did not use the application or dropped out from the trial were treated in the statistical analysis (a complete case analysis is strongly discouraged, and simple imputation techniques such as LOCF may also be problematic [4]).

	1	2	3	4	5	
subitem not at all important	○	○	○	○	○	essential

Does your paper address subitem 12a-i? *

Copy and paste relevant sections from the manuscript (include quotes in quotation marks "like this" to indicate direct quotes from your manuscript), or elaborate on this item by providing additional information not in the ms, or briefly explain why the item is not applicable/relevant for your study

"We imputed missing data using the expectation maximization and bootstrapping algorithms implemented with Amelia II in R, as no evidence emerged for unequal drop-out by condition. These imputed data sets allowed for more conservative intent-to-treat analyses than listwise deletion or last-observation carried forward and allowed us to retain high power even considering missing data. We imputed 60 data sets in accordance with the proportion of missing data for our primary outcome measure (using FASA) at 2-week follow-up."

12b) Methods for additional analyses, such as subgroup analyses and adjusted analyses

Does your paper address CONSORT subitem 12b? *

Copy and paste relevant sections from the manuscript (include quotes in quotation marks "like this" to indicate direct quotes from your manuscript), or elaborate on this item by providing additional information not in the ms, or briefly explain why the item is not applicable/relevant for your study

This subitem is not applicable for this study as we did not conduct additional analysis we did not pre-register.

X26) REB/IRB Approval and Ethical Considerations [recommended as subheading under "Methods"] (not a CONSORT item)

X26-i) Comment on ethics committee approval

	1	2	3	4	5	
subitem not at all important	○	○	○	○	○	essential

Does your paper address subitem X26-i?

Copy and paste relevant sections from the manuscript (include quotes in quotation marks "like this" to indicate direct quotes from your manuscript), or elaborate on this item by providing additional information not in the ms, or briefly explain why the item is not applicable/relevant for your study

"Study procedures were reviewed and approved by the institutional review board of the university, and informed consent was obtained from each participant on the internet prior to participation. The trial and all methods were prospectively preregistered on in ClincialTrials.gov prior to participant enrollment (NCT04453865)."

x26-ii) Outline informed consent procedures

Outline informed consent procedures e.g., if consent was obtained offline or online (how? Checkbox, etc.?), and what information was provided (see 4a-ii). See [6] for some items to be included in informed consent documents.

	1	2	3	4	5	
subitem not at all important	○	○	○	○	○	essential

Does your paper address subitem X26-ii?

Copy and paste relevant sections from the manuscript (include quotes in quotation marks "like this" to indicate direct quotes from your manuscript), or elaborate on this item by providing additional information not in the ms, or briefly explain why the item is not applicable/relevant for your study

"Eligible parents then reviewed a web-based consent form that invited them to participate."

X26-iii) Safety and security procedures

Safety and security procedures, incl. privacy considerations, and any steps taken to reduce the likelihood or detection of harm (e.g., education and training, availability of a hotline)

	1	2	3	4	5	
subitem not at all important	○	○	○	○	○	essential

Does your paper address subitem X26-iii?

Copy and paste relevant sections from the manuscript (include quotes in quotation marks "like this" to indicate direct quotes from your manuscript), or elaborate on this item by providing additional information not in the ms, or briefly explain why the item is not applicable/relevant for your study

Your answer

RESULTS

13a) For each group, the numbers of participants who were randomly assigned, received intended treatment, and were analysed for the primary outcome

NPT: The number of care providers or centers performing the intervention in each group and the number of patients treated by each care provider in each center

Does your paper address CONSORT subitem 13a? *

Copy and paste relevant sections from the manuscript (include quotes in quotation marks "like this" to indicate direct quotes from your manuscript), or elaborate on this item by providing additional information not in the ms, or briefly explain why the item is not applicable/relevant for your study

"Characteristics of the 301 participating parents and their children are shown in Table 1"

"Among parents who were assigned to the intervention condition, the majority (n=97, 66.44%) fully completed project EMPOWER, 4 (2.74%) qualified as personalized plan completers, 5 (3.42%) were psychoeducation content completers, 32 (21.92%) were partial completers, and the remaining parents (5.48%) did not begin project EMPOWER after randomization."

"We imputed missing data using the expectation maximization and bootstrapping algorithms implemented with Amelia II in R, as no evidence emerged for unequal drop-out by condition. These imputed data sets allowed for more conservative intent-to-treat analyses than listwise deletion or last-observation carried forward and allowed us to retain high power even considering missing data. We imputed 60 data sets in accordance with the proportion of missing data for our primary outcome measure (using FASA) at 2-week follow-up."

13b) For each group, losses and exclusions after randomisation, together with reasons

Does your paper address CONSORT subitem 13b? (NOTE: Preferably, this is shown in a CONSORT flow diagram) *

Copy and paste relevant sections from the manuscript (include quotes in quotation marks "like this" to indicate direct quotes from your manuscript), or elaborate on this item by providing additional information not in the ms, or briefly explain why the item is not applicable/relevant for your study

We did not exclude participants after randomization and dealt with attrition via imputations: "We imputed missing data using the expectation maximization and bootstrapping algorithms implemented with Amelia II in R, as no evidence emerged for unequal drop-out by condition. These imputed data sets allowed for more conservative intent-to-treat analyses than listwise deletion or last-observation carried forward and allowed us to retain high power even considering missing data. We imputed 60 data sets in accordance with the proportion of missing data for our primary outcome measure (using FASA) at 2-week follow-up."

13b-i) Attrition diagram

Strongly recommended: An attrition diagram (e.g., proportion of participants still logging in or using the intervention/comparator in each group plotted over time, similar to a survival curve) or other figures or tables demonstrating usage/dose/engagement.

	1	2	3	4	5	
subitem not at all important	○	○	○	○	○	essential

Does your paper address subitem 13b-i?

Copy and paste relevant sections from the manuscript or cite the figure number if applicable (include quotes in quotation marks "like this" to indicate direct quotes from your manuscript), or elaborate on this item by providing additional information not in the ms, or briefly explain why the item is not applicable/relevant for your study

Your answer

14a) Dates defining the periods of recruitment and follow-up

Does your paper address CONSORT subitem 14a? *

Copy and paste relevant sections from the manuscript (include quotes in quotation marks "like this" to indicate direct quotes from your manuscript), or elaborate on this item by providing additional information not in the ms, or briefly explain why the item is not applicable/relevant for your study

"Study recruitment began in July 2020 and ended in August 2020 once the target number of participants was achieved."

14a-i) Indicate if critical “secular events” fell into the study period

Indicate if critical “secular events” fell into the study period, e.g., significant changes in Internet resources available or “changes in computer hardware or Internet delivery resources”

	1	2	3	4	5	
subitem not at all important	○	○	○	○	○	essential

Does your paper address subitem 14a-i?

Copy and paste relevant sections from the manuscript (include quotes in quotation marks "like this" to indicate direct quotes from your manuscript), or elaborate on this item by providing additional information not in the ms, or briefly explain why the item is not applicable/relevant for your study

Your answer

14b) Why the trial ended or was stopped (early)

Does your paper address CONSORT subitem 14b? *

Copy and paste relevant sections from the manuscript (include quotes in quotation marks "like this" to indicate direct quotes from your manuscript), or elaborate on this item by providing additional information not in the ms, or briefly explain why the item is not applicable/relevant for your study

"Study recruitment began in July 2020 and ended in August 2020 once the target number of participants was achieved."

15) A table showing baseline demographic and clinical characteristics for each group

NPT: When applicable, a description of care providers (case volume, qualification, expertise, etc.) and centers (volume) in each group

Does your paper address CONSORT subitem 15? *

Copy and paste relevant sections from the manuscript (include quotes in quotation marks "like this" to indicate direct quotes from your manuscript), or elaborate on this item by providing additional information not in the ms, or briefly explain why the item is not applicable/relevant for your study

The table that describes baseline demographics and clinical characteristics can be found on page 6 with the title:

"Table 1. Sample characteristics."

15-i) Report demographics associated with digital divide issues

In ehealth trials it is particularly important to report demographics associated with digital divide issues, such as age, education, gender, social-economic status, computer/Internet/ehealth literacy of the participants, if known.

	1	2	3	4	5	
subitem not at all important	○	○	○	○	○	essential

Does your paper address subitem 15-i? *

Copy and paste relevant sections from the manuscript (include quotes in quotation marks "like this" to indicate direct quotes from your manuscript), or elaborate on this item by providing additional information not in the ms, or briefly explain why the item is not applicable/relevant for your study

The table that describes age, education, gender, socioeconomic status, marital status, and number of children can be found on page 6 with the title:

"Table 1. Sample characteristics."

16) For each group, number of participants (denominator) included in each analysis and whether the analysis was by original assigned groups

16-i) Report multiple “denominators” and provide definitions

Report multiple “denominators” and provide definitions: Report N’s (and effect sizes) “across a range of study participation [and use] thresholds” [1], e.g., N exposed, N consented, N used more than x times, N used more than y weeks, N participants “used” the intervention/comparator at specific pre-defined time points of interest (in absolute and relative numbers per group). Always clearly define “use” of the intervention.

	1	2	3	4	5	
subitem not at all important	○	○	○	○	○	essential

Does your paper address subitem 16-i? *

Copy and paste relevant sections from the manuscript (include quotes in quotation marks "like this" to indicate direct quotes from your manuscript), or elaborate on this item by providing additional information not in the ms, or briefly explain why the item is not applicable/relevant for your study

"Among parents who were assigned to the intervention condition, the majority (n=97, 66.44%) fully completed project EMPOWER, 4 (2.74%) qualified as personalized plan completers, 5 (3.42%) were psychoeducation content completers, 32 (21.92%) were partial completers, and the remaining parents (5.48%) did not begin project EMPOWER after randomization"

"First, although the completely web-based study design allowed for a large sample size and rapid, low-cost recruitment through social media, the lack of monetary compensation likely contributed to substantial attrition at follow-up (61.13%), despite scheduled email reminders."

16-ii) Primary analysis should be intent-to-treat

Primary analysis should be intent-to-treat, secondary analyses could include comparing only “users”, with the appropriate caveats that this is no longer a randomized sample (see 18-i).

	1	2	3	4	5	
subitem not at all important	○	○	○	○	○	essential

Does your paper address subitem 16-ii?

Copy and paste relevant sections from the manuscript (include quotes in quotation marks "like this" to indicate direct quotes from your manuscript), or elaborate on this item by providing additional information not in the ms, or briefly explain why the item is not applicable/relevant for your study

"We imputed missing data using the expectation maximization and bootstrapping algorithms implemented with Amelia II in R, as no evidence emerged for unequal drop-out by condition. These imputed data sets allowed for more conservative intent-to-treat analyses than listwise deletion or last-observation carried forward and allowed us to retain high power even considering missing data. We imputed 60 data sets in accordance with the proportion of missing data for our primary outcome measure (using FASA) at 2-week follow-up."

17a) For each primary and secondary outcome, results for each group, and the estimated effect size and its precision (such as 95% confidence interval)

Does your paper address CONSORT subitem 17a? *

Copy and paste relevant sections from the manuscript (include quotes in quotation marks "like this" to indicate direct quotes from your manuscript), or elaborate on this item by providing additional information not in the ms, or briefly explain why the item is not applicable/relevant for your study

"Parents assigned to the project EMPOWER condition reported significantly greater reductions in the accommodation of their children's anxiety (between-group ds=0.61; P<.001), as well as significantly greater improvements in distress tolerance (dav=0.17; between-group ds=0.43; P<.001) from baseline to 2-week follow-up, relative to control-group parents. Table 2 provides additional details regarding the multiple linear regression approach."

"Regarding within-group effects, parents who participated in project EMPOWER reported significant 2-week reductions in accommodation of child anxiety (project EMPOWER within-group dav=0.67), whereas those who were assigned to the control condition did not (control within-group dav=0.17). Between- and within-group effect sizes (dav and ds) and 95% CIs are reported in Table 3."

17a-i) Presentation of process outcomes such as metrics of use and intensity of use

In addition to primary/secondary (clinical) outcomes, the presentation of process outcomes such as metrics of use and intensity of use (dose, exposure) and their operational definitions is critical. This does not only refer to metrics of attrition (13-b) (often a binary variable), but also to more continuous exposure metrics such as "average session length". These must be accompanied by a technical description how a metric like a "session" is defined (e.g., timeout after idle time) [1] (report under item 6a).

	1	2	3	4	5	
subitem not at all important	○	○	○	○	○	essential

Does your paper address subitem 17a-i?

Copy and paste relevant sections from the manuscript (include quotes in quotation marks "like this" to indicate direct quotes from your manuscript), or elaborate on this item by providing additional information not in the ms, or briefly explain why the item is not applicable/relevant for your study

"Project EMPOWER (freely available for anonymous completion on the project's website [36]) is a web-based, self-guided SSI for parents that takes 20-30 minutes to complete."

"Among parents who were assigned to the intervention condition, the majority (n=97, 66.44%) fully completed project EMPOWER, 4 (2.74%) qualified as personalized plan completers, 5 (3.42%) were psychoeducation content completers, 32 (21.92%) were partial completers, and the remaining parents (5.48%) did not begin project EMPOWER after randomization."

17b) For binary outcomes, presentation of both absolute and relative effect sizes is recommended

Does your paper address CONSORT subitem 17b? *

Copy and paste relevant sections from the manuscript (include quotes in quotation marks "like this" to indicate direct quotes from your manuscript), or elaborate on this item by providing additional information not in the ms, or briefly explain why the item is not applicable/relevant for your study

Binary outcomes are not applicable in this study.

18) Results of any other analyses performed, including subgroup analyses and adjusted analyses, distinguishing pre-specified from exploratory

Does your paper address CONSORT subitem 18? *

Copy and paste relevant sections from the manuscript (include quotes in quotation marks "like this" to indicate direct quotes from your manuscript), or elaborate on this item by providing additional information not in the ms, or briefly explain why the item is not applicable/relevant for your study

Subgroup analyses and exploratory analysis are not applicable to this study. All analysis were pre-registered.

18-i) Subgroup analysis of comparing only users

A subgroup analysis of comparing only users is not uncommon in ehealth trials, but if done, it must be stressed that this is a self-selected sample and no longer an unbiased sample from a randomized trial (see 16-iii).

	1	2	3	4	5	
subitem not at all important	○	○	○	○	○	essential

Does your paper address subitem 18-i?

Copy and paste relevant sections from the manuscript (include quotes in quotation marks "like this" to indicate direct quotes from your manuscript), or elaborate on this item by providing additional information not in the ms, or briefly explain why the item is not applicable/relevant for your study

Your answer

19) All important harms or unintended effects in each group

(for specific guidance see CONSORT for harms)

Does your paper address CONSORT subitem 19? *

Copy and paste relevant sections from the manuscript (include quotes in quotation marks "like this" to indicate direct quotes from your manuscript), or elaborate on this item by providing additional information not in the ms, or briefly explain why the item is not applicable/relevant for your study

Harms or unintended effects were not directly assessed in the study. However, neither of the qualitative nor quantitative assessment of the intervention's acceptability included negative feedback.

19-i) Include privacy breaches, technical problems

Include privacy breaches, technical problems. This does not only include physical “harm” to participants, but also incidents such as perceived or real privacy breaches [1], technical problems, and other unexpected/unintended incidents. “Unintended effects” also includes unintended positive effects [2].

	1	2	3	4	5	
subitem not at all important	○	○	○	○	○	essential

Does your paper address subitem 19-i?

Copy and paste relevant sections from the manuscript (include quotes in quotation marks "like this" to indicate direct quotes from your manuscript), or elaborate on this item by providing additional information not in the ms, or briefly explain why the item is not applicable/relevant for your study

Your answer

19-ii) Include qualitative feedback from participants or observations from staff/researchers

Include qualitative feedback from participants or observations from staff/researchers, if available, on strengths and shortcomings of the application, especially if they point to unintended/unexpected effects or uses. This includes (if available) reasons for why people did or did not use the application as intended by the developers.

	1	2	3	4	5	
subitem not at all important	○	○	○	○	○	essential

Does your paper address subitem 19-ii?

Copy and paste relevant sections from the manuscript (include quotes in quotation marks "like this" to indicate direct quotes from your manuscript), or elaborate on this item by providing additional information not in the ms, or briefly explain why the item is not applicable/relevant for your study

Your answer

DISCUSSION

22) Interpretation consistent with results, balancing benefits and harms, and considering other relevant evidence

NPT: In addition, take into account the choice of the comparator, lack of or partial blinding, and unequal expertise of care providers or centers in each group

22-i) Restate study questions and summarize the answers suggested by the data, starting with primary outcomes and process outcomes (use)

Restate study questions and summarize the answers suggested by the data, starting with primary outcomes and process outcomes (use).

	1	2	3	4	5	
subitem not at all important	○	○	○	○	○	essential

Does your paper address subitem 22-i? *

Copy and paste relevant sections from the manuscript (include quotes in quotation marks "like this" to indicate direct quotes from your manuscript), or elaborate on this item by providing additional information not in the ms, or briefly explain why the item is not applicable/relevant for your study

"Our results support the short-term efficacy and acceptability of project EMPOWER: a self-guided, web-based SSI designed to reduce parental accommodation of child anxiety. Compared to a psychoeducational control, project EMPOWER yielded significant reductions in clinically relevant outcomes—parental accommodation and distress tolerance—across a 2-week follow-up period."

22-ii) Highlight unanswered new questions, suggest future research

Highlight unanswered new questions, suggest future research.

	1	2	3	4	5	
subitem not at all important	○	○	○	○	○	essential

Does your paper address subitem 22-ii?

Copy and paste relevant sections from the manuscript (include quotes in quotation marks "like this" to indicate direct quotes from your manuscript), or elaborate on this item by providing additional information not in the ms, or briefly explain why the item is not applicable/relevant for your study

Your answer

20) Trial limitations, addressing sources of potential bias, imprecision, and, if relevant, multiplicity of analyses

20-i) Typical limitations in ehealth trials

Typical limitations in ehealth trials: Participants in ehealth trials are rarely blinded. Ehealth trials often look at a multiplicity of outcomes, increasing risk for a Type I error. Discuss biases due to non-use of the intervention/usability issues, biases through informed consent procedures, unexpected events.

	1	2	3	4	5	
subitem not at all important	○	○	○	○	○	essential

Does your paper address subitem 20-i? *

Copy and paste relevant sections from the manuscript (include quotes in quotation marks "like this" to indicate direct quotes from your manuscript), or elaborate on this item by providing additional information not in the ms, or briefly explain why the item is not applicable/relevant for your study

"Several limitations of this trial warrant discussion and suggest directions for future studies. First, although the completely web-based study design allowed for a large sample size and rapid, low-cost recruitment through social media, the lack of monetary compensation likely contributed to substantial attrition at follow-up (61.13%), despite scheduled email reminders. However, it is worth noting that offering greater monetary compensation may have introduced additional bias to the sample selection. This limitation was addressed via a rigorous missing data approach, which has shown utility with high rates of missing data, including those observed in this trial [34]. Second, similar to the limitations noted in much of the literature on parenting interventions, the homogeneity in sex (98% mothers), race and ethnicity, and education status in our sample limited the generalizability of our results across diverse groups of parents. This may be due to the selection bias introduced by recruitment through social media as Facebook likely distributed the advertisements to users who are interested in the study topic. As the study team did not have control over the algorithms that are used to distribute the advertisement, it limited our ability to reach a more diverse population. Moving forward, it will be critical to test the acceptability and effects of project EMPOWER and other self-guided SSIs among members of marginalized and minoritized communities of individuals who are systematically least likely to access traditional, face-to-face mental health treatments owing to financial, logistic, and stigma-related barriers.

Given that non–English-speaking parents were unable to take part in the study (project EMPOWER is currently available only in English), efforts to translate project EMPOWER into various languages may greatly facilitate tests of its acceptability and utility among more diverse caregivers. Third, because this trial was the first to assess the acceptability and proximal effects of project EMPOWER, we included a relatively brief 2-week follow-up period. Thus, results address only the short-term effects of the intervention on known risk factors for child anxiety. Given that some trials of self-guided SSIs have demonstrated clinical benefits for youth up to 9 months after the intervention [30,35], the longer-term effects of project EMPOWER remain important to explore. Such studies may investigate whether the intervention can prevent the emergence of child anxiety symptoms and evaluate improvements in parent accommodation and distress tolerance as possible change mechanisms."

21) Generalisability (external validity, applicability) of the trial findings

NPT: External validity of the trial findings according to the intervention, comparators, patients, and care providers or centers involved in the trial

21-i) Generalizability to other populations

Generalizability to other populations: In particular, discuss generalizability to a general Internet population, outside of a RCT setting, and general patient population, including applicability of the study results for other organizations

	1	2	3	4	5	
subitem not at all important	○	○	○	○	○	essential

Does your paper address subitem 21-i?

Copy and paste relevant sections from the manuscript (include quotes in quotation marks "like this" to indicate direct quotes from your manuscript), or elaborate on this item by providing additional information not in the ms, or briefly explain why the item is not applicable/relevant for your study

"Similar to the limitations noted in much of the literature on parenting interventions, the homogeneity in sex (98% mothers), race and ethnicity, and education status in our sample limited the generalizability of our results across diverse groups of parents. This may be due to the selection bias introduced by recruitment through social media as Facebook likely distributed the advertisements to users who are interested in the study topic. As the study team did not have control over the algorithms that are used to distribute the advertisement, it limited our ability to reach a more diverse population. Moving forward, it will be critical to test the acceptability and effects of project EMPOWER and other self-guided SSIs among members of marginalized and minoritized communities of individuals who are systematically least likely to access traditional, face-to-face mental health treatments owing to financial, logistic, and stigma-related barriers."

"Given that non–English-speaking parents were unable to take part in the study (project EMPOWER is currently available only in English), efforts to translate project EMPOWER into various languages may greatly facilitate tests of its acceptability and utility among more diverse caregivers"

21-ii) Discuss if there were elements in the RCT that would be different in a routine application setting

Discuss if there were elements in the RCT that would be different in a routine application setting (e.g., prompts/reminders, more human involvement, training sessions or other co-interventions) and what impact the omission of these elements could have on use, adoption, or outcomes if the intervention is applied outside of a RCT setting.

	1	2	3	4	5	
subitem not at all important	○	○	○	○	○	essential

Does your paper address subitem 21-ii?

Copy and paste relevant sections from the manuscript (include quotes in quotation marks "like this" to indicate direct quotes from your manuscript), or elaborate on this item by providing additional information not in the ms, or briefly explain why the item is not applicable/relevant for your study

Your answer

OTHER INFORMATION

23) Registration number and name of trial registry

Does your paper address CONSORT subitem 23? *

Copy and paste relevant sections from the manuscript (include quotes in quotation marks "like this" to indicate direct quotes from your manuscript), or elaborate on this item by providing additional information not in the ms, or briefly explain why the item is not applicable/relevant for your study

"ClinicalTrials.gov NCT04453865; https://clinicaltrials.gov/ct2/show/NCT04453865?term=NCT04453865&draw=2&rank=1"

24) Where the full trial protocol can be accessed, if available

Does your paper address CONSORT subitem 24? *

Cite a Multimedia Appendix, other reference, or copy and paste relevant sections from the manuscript (include quotes in quotation marks "like this" to indicate direct quotes from your manuscript), or elaborate on this item by providing additional information not in the ms, or briefly explain why the item is not applicable/relevant for your study

"The entire preregistration can be found on ClinicalTrials.gov (NCT04453865). Deidentified data and code for all preregistered analyses are available on the Open Science Framework [37]."

25) Sources of funding and other support (such as supply of drugs), role of funders

Does your paper address CONSORT subitem 25? *

Copy and paste relevant sections from the manuscript (include quotes in quotation marks "like this" to indicate direct quotes from your manuscript), or elaborate on this item by providing additional information not in the ms, or briefly explain why the item is not applicable/relevant for your study

"This study was funded by a graduate research grant from the Psi Chi Honor Society. JYS receives funding from the Health Policy Research Scholars Program. JLS receives research support from the National Institutes of Health (DP5OD28123), the Klingenstein Third Generation Foundation, the American Psychological Foundation, the Upswing Fund, and Limbix, Inc. JLS is also a coauthor of a therapeutic workbook for the youth (published by New Harbinger Publications) and a coeditor of a book on low-intensity mental health interventions for the youth (published by Oxford University Press)."

X27) Conflicts of Interest (not a CONSORT item)

X27-i) State the relation of the study team towards the system being evaluated

In addition to the usual declaration of interests (financial or otherwise), also state the relation of the study team towards the system being evaluated, i.e., state if the authors/evaluators are distinct from or identical with the developers/sponsors of the intervention.

	1	2	3	4	5	
subitem not at all important	○	○	○	○	○	essential

Does your paper address subitem X27-i?

Copy and paste relevant sections from the manuscript (include quotes in quotation marks "like this" to indicate direct quotes from your manuscript), or elaborate on this item by providing additional information not in the ms, or briefly explain why the item is not applicable/relevant for your study

"Conflicts of Interest
None declared."

About the CONSORT EHEALTH checklist

As a result of using this checklist, did you make changes in your manuscript? *

- ( ) yes, major changes
- ( ) yes, minor changes
- (•) no

What were the most important changes you made as a result of using this checklist?

Your answer

How much time did you spend on going through the checklist INCLUDING making changes in your manuscript *

4-5 hours

Your answer must have a minimum of 25 characters.

As a result of using this checklist, do you think your manuscript has improved? *

- ( ) yes
- (•) no
- ( ) Other:

Would you like to become involved in the CONSORT EHEALTH group?

This would involve for example becoming involved in participating in a workshop and writing an "Explanation and Elaboration" document

- ( ) yes
- (•) no
- ( ) Other:

Clear selection

Any other comments or questions on CONSORT EHEALTH

Your answer

STOP - Save this form as PDF before you click submit

To generate a record that you filled in this form, we recommend to generate a PDF of this page (on a Mac, simply select "print" and then select "print as PDF") before you submit it.

When you submit your (revised) paper to JMIR, please upload the PDF as supplementary file.

Don't worry if some text in the textboxes is cut off, as we still have the complete information in our database. Thank you!

Final step: Click submit !

Click submit so we have your answers in our database!

Submit

Never submit passwords through Google Forms.

This content is neither created nor endorsed by Google. Report Abuse - Terms of Service - Privacy Policy

Google Forms
